# Intracellular formate modulates a motility-invasion switch in *Salmonella* Typhimurium

**DOI:** 10.1371/journal.ppat.1013453

**Published:** 2025-09-04

**Authors:** Debapriya Mukherjee, Salik Noor, Tamoghna Mukherjee, Mahavir Singh, Dipshikha Chakravortty

**Affiliations:** 1 Department of Microbiology and Cell Biology, Division of Biological Sciences, Indian Institute of Science, Bangalore, India; 2 Molecular Biophysics Unit, Indian Institute of Science, Bangalore, India; 3 School of Biology, Indian Institute of Science Education and Research, Thiruvananthapuram, India; INSERM U1220, FRANCE

## Abstract

Host-derived short-chain fatty acids (SCFAs) are essential for *Salmonella* Typhimurium (STM) virulence. Formate, an SCFA found in the ileum, enhances STM invasion, but the role of the intracellular formate pool in STM pathogenesis remains poorly understood. Deletion of the *pflB* gene, which encodes pyruvate-formate lyase, depletes this intracellular pool, leading to reduced flagellation and increased expression of pathogenicity island-1 genes (*hilA* and *prgH*). This response is driven by elevated intracellular pH and membrane damage, triggering a shift from adhesion to invasion. This transition is regulated by the membrane-bound extra cytoplasmic sigma factor RpoE via the CsrA/*csrB* pathway. Replenishing the intracellular formate pool enabled STM Δ*pflB* to use formate as a signalling molecule to modulate virulence. Our findings underscore the critical role of intracellular formate in maintaining pH balance and coordinating the regulation of flagellar and SPI-1 genes, emphasizing the need to fine-tune *pflB* expression across intestinal regions for optimal STM invasion.

## Author summary

The ability of *Salmonella* Typhimurium to modulate the expression of its virulence factors in response to diverse environmental cues has positioned it as a highly successful pathogen. However, the role of metabolite regulation during various stages of its pathogenic lifecycle, as well as its connection to the control of *S.* Typhimurium (STM) pathogenesis, remains poorly understood. In this study, we investigated the role of the metabolite formate in the pathogenic lifestyle of STM. Our results demonstrate that a reduction in intracellular formate levels results in a decreased number of flagella and reduced motility, accompanied by increased expression of SPI-1 genes *hilA* and *prgH*. This process is driven by membrane damage caused by formate depletion, which induces the expression of the small RNA *csrB* through the heightened activity of the extra-cytoplasmic sigma factor RpoE. These findings provide critical insights into the metabolic regulation of the invasion process in STM. Furthermore, this study paves the way for exploring similar metabolic and regulatory pathways in other bacterial species, potentially identifying conserved strategies employed by pathogens to adapt and persist within their hosts.

## Introduction

The genus *Salmonella* consists of Gram-negative gammaproteobacteria that infect a wide range of human and animal hosts [1]. Non-typhoidal serovars, such as *Salmonella* Typhimurium, are commonly associated with self-limiting diarrheal illness with relatively low fatality. According to the Global Burden of Diseases, Injuries, and Risk Factors Study (GBD) 2017, there were an estimated 95.1 million cases of *Salmonella* enterocolitis worldwide, resulting in 50,771 deaths [2–4]. Although non-typhoidal infections are typically confined to the intestines, these serovars can occasionally invade sterile tissues, leading to bacteraemia, meningitis, and other focal infections, which significantly increase mortality rates.[5].

*Salmonella* Typhimurium enters the host via contaminated food and water. One of the defence mechanisms in the host is the presence of short-chain fatty acids (SCFAs) in the small intestine. SCFAs are produced through the anaerobic fermentation of non-digestible polysaccharides, such as resistant starches and dietary fibres. The SCFAs found in the intestine—formate (C1), acetate (C2), propionate (C3), and butyrate (C4), with concentrations reaching up to 10–100 mM. Recent studies have extensively explored the role of SCFAs ((like butyrate and propionate) in *Salmonella* infections [6–10]. For example, butyrate downregulates the expression of pathogenicity island-1 (SPI-1) genes in *S.* Typhimurium, thereby reducing bacterial invasion and translocation from the intestines to the bloodstream [11–13].

Formate present in the intestine has been previously shown to act as a diffusible signal promoting *Salmonella* Typhimurium invasion [14]. Imported, unmetabolized formic acid functions as a cytoplasmic signal by binding to HilD, the master transcriptional regulator of *Salmonella* invasion, thereby preventing inhibitory fatty acids from binding and extending the activity of HilD, which leads to the derepression of invasion genes [14]. Moreover, formate utilization by respiratory enzymes such as formate dehydrogenase-N (FdnGHI) and formate dehydrogenase-O (FdoGHI) plays a key role in *Salmonella* colonization of the gut [15]. *S.* Typhimurium can source formate from both its own pyruvate-formate lyase (PflB) and from the intestinal environment [16,17]. However, the role of endogenously produced formate in regulating *Salmonella* virulence and pathogenesis have seldom been reported [18].

In this study, we examined the role of endogenously produced formate in the pathogenic lifestyle of *S.* Typhimurium. To the best of our knowledge, we are the first to identify the role of the *pflB* gene in regulating cytosolic pH of STM, which is instrumental in maintaining the flagellar synthesis. Quite interestingly, in the wild-type strain with an intact formate pool, extracellular formate functions as a signalling molecule, highlighting the distinct roles of intracellular and extracellular formate pools in regulating *Salmonella* virulence. We demonstrate that regulating the *pflB* gene in various regions of the intestine may be a strategy utilized by *Salmonella* to optimize its invasion of the distal ileum, its primary target site.

## Results

### The deletion of the *pflB* gene led to decreased invasion efficiency in the Caco-2 cell line, which corresponded to a reduced organ burden in C57BL/6 mice five days after infection through oral gavaging

To investigate the role of endogenous formate levels in *Salmonella* virulence and pathogenesis, we created knockout strains of *pflB* and *focA* using the one-step gene inactivation technique outlined by Datsenko and Wanner [19]. FocA is the formate transporter in *Salmonella* Typhimurium that functions as a passive export channel at high external pH and switches to an active importer of formate at low pH [20]. The deletion of either gene did not impact the *in vitro* growth of *Salmonella* in either complex LB media or M9 Minimal Media (S1A–S1B Fig). Since the *focA* and *pflB* genes are part of the same operon, we investigated whether deleting one gene affected the expression of the other [21]. Our study revealed that deleting the upstream *focA* gene resulted in decreased expression of the downstream *pflB* gene (S1C Fig). However, deletion of *pflB* increased the expression of *focA* (S1D Fig).

**Fig 1:**
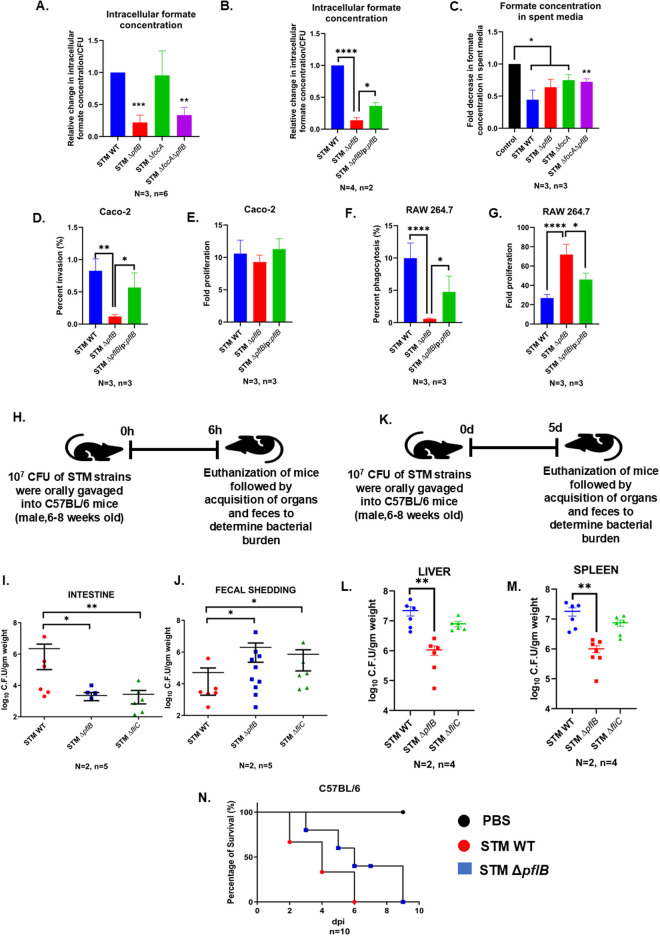
The deletion of the *pflB* gene led to decreased invasion efficiency in the Caco-2 cell line, which corresponded to a reduced organ burden in C57BL/6 mice five days after infection through oral gavaging. A. GC-MS mediated quantification of relative intracellular formate in STM WT, STM Δ*pflB*, STM Δ*focA*, STM Δ*focA*Δ*pflB*. Data is represented as Mean + /-SEM of N = 3, n = 6. B. GC-MS assisted quantification of relative intracellular formate in STM WT, STM Δ*pflB*, STM Δ*pflB/* pQE60:*pflB*. Data is represented as Mean + /-SEM of N = 4, n = 2. C. GC-MS mediated quantification of residual formate in the spent media post incubation by STM WT, STM Δ*pflB*, STM Δ*focA*, STM Δ*focA*Δ*pflB*. Data is represented as Mean + /-SEM of N = 3, n = 3. D. Percent invasion of STM WT, STM Δ*pflB*, and STM Δ*pflB/* pQE60:*pflB* in Caco-2 cell line. Data is represented as Mean + /-SEM of N = 3, n = 3. E. Fold proliferation of STM WT, STM *Δ*pflB**, and STM Δ*pflB/* pQE60:*pflB* in Caco-2 cell line. Data is represented as Mean + /-SEM of N = 3, n = 3. F. Percent phagocytosis of STM WT, STM Δ*pflB*, and STM Δ*pflB/* pQE60:*pflB* in RAW 264.7 cell line. Data is represented as Mean + /-SEM of N = 3, n = 3. G. Fold proliferation of STM WT, STM *Δ*pflB**, and STM Δ*pflB/* pQE60:*pflB* in RAW 264.7 cell line. Data is represented as Mean + /-SEM of N = 3, n = 3. H. Schematic showing the protocol followed for invasion assay in C57BL/6 mice. I. Bacterial burden in the intestine post 6h of infecting the mice with STM WT, STM Δ*pflB*, and STM Δ*fliC* via oral gavaging. Data is represented as Mean + /-SEM of N = 2, n = 5. J. Bacterial burden in the feces of mice post 6h of infection with STM WT, STM Δ*pflB*, and STM Δ*fliC* via oral gavaging. Data is represented as Mean + /-SEM of N = 2, n = 5. K. Schematic showing the protocol followed for determining the organ burden of STM WT, STM Δ*pflB,* and STM Δ*fliC* 5 days post oral gavaging. L. Organ burden of STM WT, STM Δ*pflB*, and STM Δ*fliC* in liver 5 days post oral gavaging. Data is represented as Mean + /-SEM of N = 2, n = 4. M. Organ burden of STM WT, STM Δ*pflB*, and STM Δ*fliC* in spleen 5 days post oral gavaging. Data is represented as Mean + /-SEM of N = 2, n = 4. N. Survival rates of C57BL/6 mice post infection with STM WT and STM Δ*pflB* through oral route. (Unpaired two-tailed Student’s t-test for column graphs, Two-way ANOVA for grouped data, Mann-Whitney U-test for animal experiment data (**** p < 0.0001, *** p < 0.001, ** p < 0.01, * p < 0.05)).

To confirm that deleting *pflB* and *focA* indeed depleted the native formate pool of the cell, we collected lysates from logarithmic phase cultures of all strains and quantified the formate concentration using Gas Chromatography-Mass Spectrometry (GC-MS). After normalizing by CFU, we observed that there was a significant reduction in the relative intracellular formate concentrations upon deletion of *pflB*, whereas deletion of *focA* did not cause a notable change. There was a significantly reduced intracellular formate level in STM Δ*focA*Δ*pflB* (Fig 1A). Complementing *pflB* via an extragenous plasmid pQE60 partially recovered the intracellular formate pool in STM Δ*pflB* (Fig 1B). This finding suggested the possibility of an alternative formate transporter. To investigate further, we incubated secondary cultures of all strains in formate-supplemented media and measured formate concentration in the spent media after 3 hours by GC-MS. We found a significant reduction in formate concentration in the spent media of STM Δ*focA* compared to the control, indicating the presence of an alternative formate importer (Fig 1C). Since STM Δ*focA* retained intracellular formate levels similar to STM WT and our data suggested the presence of an alternate formate transporter, we focused our study on STM Δ*pflB*, where we could successfully deplete the intracellular formate pool.

Intracellular Cell Survival Assay (ICSA) using the human colorectal carcinoma cell line Caco-2 showed that STM Δ*pflB* exhibited reduced invasion efficiency compared to STM WT (**Fig 1D**). The invasion deficiency in STM Δ*pflB* was also rescued by complementing the gene with an exogenous pQE60 plasmid (**Fig 1D**). STM Δ*pflB* exhibited comparable proliferation to the STM WT in the Caco-2 cell line (**Fig 1E**). Although the initial absolute CFU counts for STM Δ*pflB* were lower, normalization using the 16-hour CFU values revealed a similar fold increase in bacterial numbers compared to the wild-type. This indicates that STM Δ*pflB* displayed a deficiency primarily during the early phase of infection in the Caco-2 model, but once internalized, its proliferation matched that of the WT strain. In the murine macrophage cell line RAW 264.7, STM Δ*pflB* showed a decreased phagocytosis and hyperproliferation compared to STM WT (**Fig 1F**,**1G**).

To determine if the invasion deficiency observed in STM Δ*pflB* was consistent in an animal model, we orally gavaged 10^7^ CFU of STM WT and STM Δ*pflB* into 6–8-week-old male C57BL/6 mice. Adhesion-deficient STM Δ*fliC* served as a control. Six hours post-infection, we observed a reduced bacterial load in the mouse intestine for STM Δ*pflB*, which was associated with increased faecal shedding compared to STM WT (**Fig 1H**,**1I**,**1J**). These findings were consistent with the results for the control strain STM Δ*fliC*, which is known to have reduced adhesion and is cleared more rapidly from streptomycin-treated mice and behaved similarly in our system without any antibiotic treatment [22].

To further understand the role of *pflB* deletion in overall *Salmonella* pathogenesis in *in-vivo* mouse model, we orally gavaged 10^7^ CFU of bacteria in 6–8 weeks old male C57BL/6 mice and euthanized the mice 5 days post-infection (dpi) to determine the bacterial burden in the liver, the spleen, and the brain (Fig 1K,1L,1M). We found a significantly reduced bacterial burden in the liver and the spleen when infected with STM Δ*pflB* as compared to infection with STM WT (Fig 1L,1M). However, there was no significant organ burden difference between STM WT and the adhesion and invasion deficient STM Δ*fliC* as reported previously. This was in line with previous reports regarding deletion strain of *flhD*, the master regulator of flagella synthesis, that was also deficient in invasion to epithelial cells [23]. We also found that C57BL/6 mice infected with STM Δ*pflB* had a better survival rate as compared to mice infected with STM WT (Fig 1N). We subjected C57BL/6 mice to an intra-peritoneal infection with 10^3^ CFU of both the WT and STM Δ*pflB* knockout strains. Under these conditions, we observed no significant change in the organ burden of STM WT and STM Δ*pflB* 3dpi (S2A–S2D Fig). This indicates that although *pflB* deletion may disrupt early intestinal colonization of *Salmonella*, it influences pathways involved in gastrointestinal transit of *Salmonella*, as wild-type-like infection is restored when the oral route is bypassed.

### STM Δ*pflB* was deficient in flagella and the deficiency was partially recovered upon supplementation of 40 mM sodium formate

**Fig 2:**
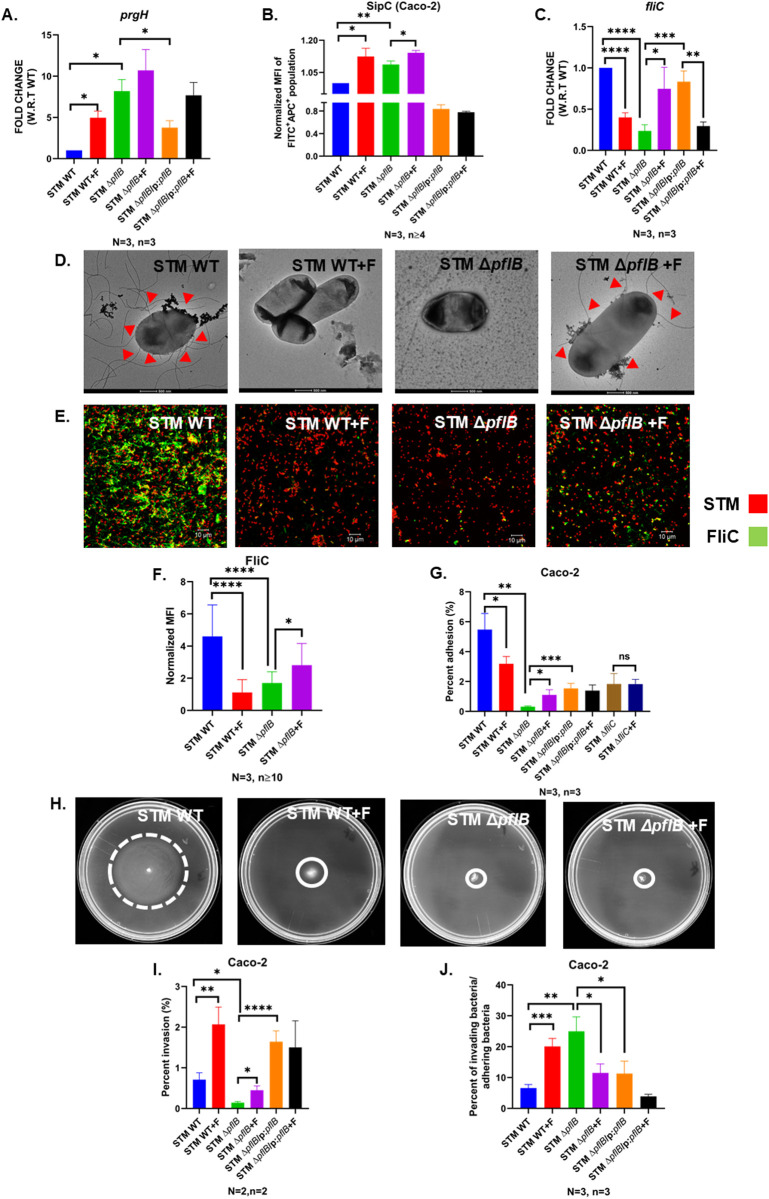
STM Δ*pflB* was deficient in flagella and the deficiency was recovered upon supplementation of formate. A. RT-qPCR mediated study of expression profile of *prgH* in the logarithmic phase cultures of STM WT, STM Δ*pflB*, and STM Δ*pflB/* pQE60:*pflB* (+/-formate). Data is represented as Mean + /-SEM of N = 3, n = 3. B. Flow cytometry assisted normalized MFI of APC (corresponding to SipC protein expression) in FITC positive (bacteria-positive) Caco-2 cells. Data is represented as Mean + /-SEM of N = 3, n ≥ 4. C. RT-qPCR mediated study of expression profile of *fliC* in the logarithmic phase cultures of STM WT, STM Δ*pflB,* and STM Δ*pflB/* pQE60:*pflB* (+/-formate). Data is represented as Mean + /-SEM of N = 2, n = 3. D. TEM assisted visualization of the flagellar structures in STM WT, STM Δ*pflB* (+/-formate). Red arrows denote flagellar structures. E. Confocal microscopy assisted visualization of the flagellar structures in STM WT, STM Δ*pflB* (+/-formate). Images are representative of N = 3, n ≥ 10. F. NormalizedMFI, i.e.,., MFI of Secondary antibody for Rabbit generated Anti-fli antibody/ MFI for bacterial mCherry in confocal microscopy assisted visualization of the flagellar structures in STM WT, STM Δ*pflB* (+/-formate). Data is representative of N = 3, n ≥ 10 and is shown as Mean + /-SD. G. Percent adhesion of STM WT, STM Δ*pflB*, STM Δ*pflB/* pQE60:*pflB,* STM Δ*fliC*(+/-formate) in Caco-2 cell line. Data is represented as Mean + /-SEM of N = 3, n = 3. H. Swim motility of STM WT and STM Δ*pflB* (+/-formate) on 0.25% Agar containing LB plates. I. Percent invasion of STM WT, STM Δ*pflB*, STM Δ*pflB/*pQE60:*pflB* (+/-formate) in Caco-2 cell line. Data is represented as Mean + /-SEM of N = 2, n = 2. J. Graphical representation of percent of invading bacteria/ adhering bacteria for STM WT, STM Δ*pflB*, STM Δ*pflB/*pQE60:*pflB* (+/-formate) in Caco-2 cell line. Data is represented as Mean + /-SEM of N = 3, n = 3. (Unpaired two-tailed Student’s t-test for column graphs, Two-way ANOVA for grouped data, Mann-Whitney U-test for animal experiment data (**** p < 0.0001, *** p < 0.001, ** p < 0.01, * p < 0.05)).

*Salmonella* Typhimurium uses adhesins, such as flagella, to attach to host cells during infection [24–26]. Following successful adhesion, it employs the *Salmonella* Pathogenicity Island-1 (SPI-1) machinery to invade non-phagocytic epithelial cells [27–29]. Our previous findings indicated that STM Δ*pflB* has reduced invasion efficiency in Caco-2 cells. We opted to proceed by supplementing exogenous formate to STM Δ*pflB* to determine whether the effects observed from depleting intracellular formate could indeed be reversed through the addition of extracellular formate. In our further studies, we used a concentration of 40 mM (indicated as F in figure panels), as it yielded maximal expression of the SPI-1 regulatory gene *hilA* in STM WT in a concentration-dependent analysis ranging from 0 to 50 mM (S3A Fig). Interestingly, we observed that STM Δ*pflB* exhibited increased expression of *prgH*, which encodes a structural protein of the SPI-1 apparatus; this expression was reduced in the complemented strain (Fig 2A). This expression was further elevated in STM *ΔpflB* +formate. Additionally, STM WT + F showed significantly higher *prgH* expression compared to STM WT, consistent with the findings of Huang *et al.* [16]. To determine whether the increased transcript levels of the SPI-1 apparatus corresponded to a functional PrgH that led to enhanced secretion of SPI-1 effectors during infection, we quantified the abundance of SipC—a SPI-1 effector—within 30 minutes of infecting Caco-2 cells [30]. Our analysis revealed that Caco-2 cells infected with STM Δ*pflB* had higher SipC levels than those infected with STM WT. Moreover, supplementation with formate further amplified SipC secretion in STM Δ*pflB*-infected cells. Similarly, cells infected with STM WT+ formate showed increased SipC secretion compared to those infected with STM WT alone (Figs 2B,S3B).

We also found that STM Δ*pflB* had lower *fliC* expression, which was partially restored under formate supplementation and partially recovered in the complemented strain. Formate supplementation reduced *fliC* expression in STM WT as well, compared to the untreated control (Fig 2C). We confirmed this via Transmission Electron Microscopy (TEM) as well, where we found that STM WT treated with formate had lesser flagella compared to untreated control. STM Δ*pflB* had a reduced flagellar number, which was partially restored upon supplementation of F (Fig 2D). Quantitative analysis of normalized median fluroscence intensity (MFI) in confocal microscopy and adhesion assays in the Caco-2 cell line further validated the findings from qRT-PCR and TEM (Figs 2E–2G,S4A). STM WT treated with formate also showed lesser motility on swim agar plate compared to STM WT, however, we did not see a recovery in motility of STM Δ*pflB* upon formate supplementation (Fig 2H). This indicates that while formate supplementation in STM Δ*pflB* may restore the transcription level of *fliC* and its protein expression, it does not lead to a recovery in flagella-mediated motility. The partially restored adhesion observed in STM Δ*pflB*+ formate in the Caco-2 cell line could be attributed to the increased surface hydrophobicity of the flagellar filaments, resulting from the recovery in protein levels. This enhanced hydrophobicity can promote stronger interactions for effective adhesion to eukaryotic host cells [31]. Further experimentation involved incubating secondary cultures of STM WT and STM Δ*pflB* in varying concentrations of formate. Notably, formate significantly inhibited *fliC* expression in STM WT starting from a concentration of 1 mM, while a concentration as low as 0.125 mM was sufficient to restore reduced *fliC* expression in STM Δ*pflB* (S4B Fig). Additionally, 10 mM formate significantly upregulated *hilA*—the master regulator of SPI-1—in both strains (S4C Fig).

F supplementation in STM Δ*pflB* was also associated with an improved invasion in Caco-2 cell line. We also found that STM WT supplemented with formate had a higher percent invasion compared to STM WT (Fig 2I). We also used confocal microscopy to visualize the greater abundance of intracellular bacteria at the same time point. LAMP-1, a marker of the *Salmonella*-containing vacuole (SCV), served as an intracellular marker (S5 Fig) [32].

While deletion of *pflB* reduced motility in *Salmonella*, it resulted in elevated SPI-1 transcript levels and increased secretion of SPI-1 effectors, which are essential for invasion. This was reflected in a higher proportion of invading bacteria relative to adhering bacteria in the STM Δ*pflB* strain. As anticipated, this ratio declined upon formate supplementation. Interestingly, formate addition to STM WT also led to an increase in this ratio, indicating that formate supplementation enhances the invasion capacity of adhering bacteria (Fig 2J).

The double knockout strain STM Δ*focA*Δ*pflB* exhibited similar growth kinetics as STM WT in LB broth and M9 Minimal Media (S6A,S6B Fig). However, due to its depleted formate pool, it was also deficient in adhesion in Caco-2 cell line and recovery was visualized when incubated with formate (S6C Fig). Infection with STM Δ*focA* with an intact formate pool led to a similar bacterial burden in the liver and the spleen as STM WT 5 dpi. STM Δ*focA*Δ*pflB* showed lesser organ burden in the liver and spleen of the C57 mice model (S6D,S6E Fig).

### STM Δ*pflB* had a higher intracellular pH and higher membrane depolarization, both of which were recovered upon F supplementation

**Fig 3:**
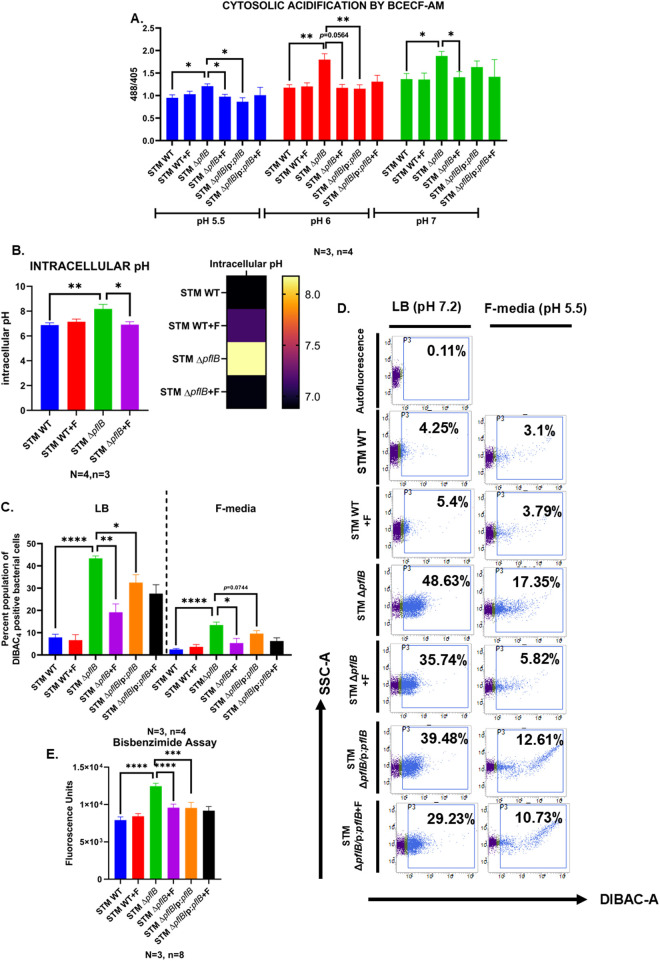
STM Δ*pflB* had a higher intracellular pH and higher membrane depolarization, both of which were recovered upon formate supplementation. A. BCECF-AM dye mediated elucidation of the relative ipH in the logarithmic cultures of STM WT, STM Δ*pflB,* STM Δ*pflB/*pQE60:*pflB* (+/-formate) in phosphate buffers of pH 5.5, 6, and 7. Data is represented as Mean + /-SEM of N = 3, n = 4. B. Elucidation of the ipH in the logarithmic cultures of STM WT/pBAD-pHuji and STM *Δ*pflB**/pBAD-pHuji (+/-formate). Data represented both as bar graph and heat map and is Mean + /-SEM of N = 4, n = 3. C. Flow cytometry obtained quantification of percent- DiBAC_4_ positive cells in the logarithmic cultures of STM WT, STM Δ*pflB,* STM Δ*pflB/*pQE60:*pflB* (+/-formate) incubated in LB media (pH 7.2) and F-media (pH 5.5). Data is represented as Mean + /-SEM of N = 3, n = 4. D. Representative FACS percent positive profiles of DiBAC_4_ staining in the logarithmic cultures of STM WT, STM Δ*pflB,* STM Δ*pflB/*pQE60:*pflB* (+/-formate) in LB and F-media. Data is representative of N = 3, n = 4. E. Bisbenzimide assay to quantify the membrane damage in the logarithmic cultures of STM WT, STM Δ*pflB,* STM Δ*pflB/*pQE60:*pflB* (+/-formate). Data is represented as Mean + /-SEM of N = 3, n = 8. (Unpaired two-tailed Student’s t-test for column graphs, Two-way ANOVA for grouped data, Mann-Whitney U-test for animal experiment data (**** p < 0.0001, *** p < 0.001, ** p < 0.01, * p < 0.05)).

Previous studies in *Escherichia coli* indicated that formate translocation through the FocA channel and its metabolism via the FHL-1 (Formate Hydrogen Lyase-1) complex are crucial for maintaining cellular pH homeostasis during anaerobic growth or fermentation [33–39]. This raised the question of whether formate synthesis during aerobic growth in *Salmonella* Typhimurium could also contribute to maintaining intracellular pH (ipH). Additionally, weak acids like acetate and benzoate can cross the cell membrane in their neutral forms and subsequently dissociate, thereby reducing ipH [40]. Based on this, we also hypothesized that the endogenous formate pool might play a role in maintaining ipH. To investigate how changes in the surrounding media’s pH affect ipH, we incubated STM WT and STM Δ*pflB* (+/- formate) in phosphate buffers at pH levels 5.5, 6, and 7. After incubation, we stained the cells with the pH-sensitive dye BCECF-AM. A higher 488/405 nm fluorescence ratio for STM Δ*pflB* under all pH conditions indicated a higher ipH in the knockout strain compared to the WT. Complementation of *pflB* in the knockout strain partially reduced the 488/405 ratio at pH 7, with a more pronounced decrease observed at lower pH values of 5.5 and 6 (**Fig 3A**).

To further confirm these results, we transformed STM WT and STM Δ*pflB* with the plasmid pBAD-pHuji, which encodes a pH-sensitive red fluorescent protein. The higher ipH observed in STM Δ*pflB*/pBAD-pHuji confirmed that *pflB* plays a significant role in maintaining cytosolic pH in *Salmonella* Typhimurium. Consistent with the BCECF-AM staining results, formate did not affect the ipH of STM WT/pBAD-pHuji (**Fig 3B**). In the heat map displaying mean values, the STM WT/pHuji strain supplemented with formate shows a shift toward violet, suggesting a potential increase in intracellular pH with formate treatment. However, as indicated by the bar graph, this change is not statistically significant. These findings suggest the possibility that extracellular and intracellular formate pools might independently regulate the expression of *fliC* and *hilA* through distinct mechanisms.

One of the causes of membrane depolarization in biological systems is disrupted pH homeostasis [41]. To investigate membrane potential in all strains with and without formate supplementation, we analysed the exponential cultures of STM Δ*pflB* in LB. These cultures showed a higher population of DiBAC_4_-positive cells, indicating increased membrane depolarization. Supplementation with formate successfully reduced membrane depolarization in STM Δ*pflB*, but it had no effect on membrane polarization in STM WT. STM Δ*pflB*/pQE60: *pflB* had lesser DiBAC_4_ positive bacterial cells compared to STM Δ*pflB* (Fig 3C,3D). Furthermore, the extent of membrane depolarization in STM Δ*pflB* was reduced when stationary-phase bacteria were subcultured into acidic F-media (pH 5) (Fig 3C). The H⁺ ions in the acidic F-media may help counteract the disrupted cytosolic acidification in the *pflB* knockout strain, thereby restoring membrane polarization. The higher membrane damage in STM Δ*pflB* was also quantified with the DNA-binding fluorescent dye bisbenzimide (Fig 3E). We further confirmed that membrane depolarization in STM Δ*pflB* was unrelated to flagellar deficiency by comparing it with a *fliC* deletion mutant (S7 Fig).

These findings suggest that the intracellular formate pool is crucial for maintaining intracellular pH (ipH) in *Salmonella* Typhimurium, which in turn supports cell membrane polarization. We also explored whether the elevated ipH in the *pflB* knockout strain contributes to disrupted flagellar apparatus synthesis. Incubation of STM Δ*pflB* in acidic LB medium (pH 6) led to an increase in the *fliC* transcript level (S8A Fig), indicating that higher ipH is responsible for these downstream effects.

### STM Δ*pflB* had a higher transcript level of *csrB*, raising the possibility of flagella inhibition via the *csr* sRNA system

**Fig 4:**
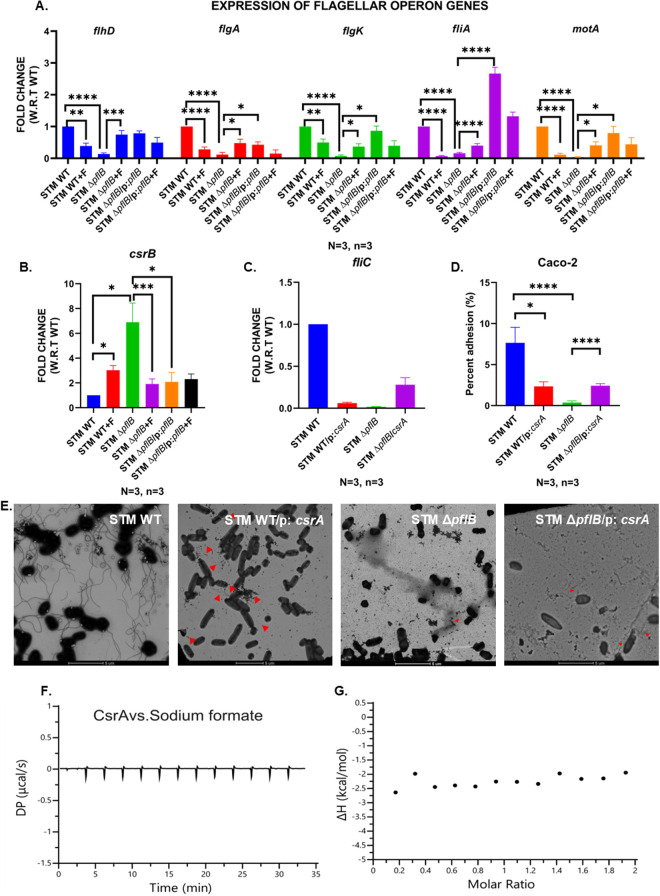
STM Δ*pflB* had a higher transcript level of *csrB*, raising the possibility of flagella inhibition via the *csr* sRNA system. A. RT-qPCR mediated expression profile of the flagellar operon genes *flhD*, *flgA*, *flgK*, *fliA*, *motA* in the logarithmic cultures of STM WT, STM Δ*pflB,* STM Δ*pflB/*pQE60:*pflB* (+/-formate)*.* Data is represented as Mean + /-SEM of N = 3, n = 3. B. RT-qPCR mediated expression profile of the sRNA *csrB* in the logarithmic cultures of STM WT, STM Δ*pflB,* STM Δ*pflB/*pQE60:*pflB* (+/-formate). Data is represented as Mean + /-SEM of N = 3, n = 3. C. RT-qPCR mediated expression profile of the *fliC* in the logarithmic cultures of STM WT and STM Δ*pflB* transformed with an pQE60*: csrA* under an IPTG-inducible system. Data is represented as Mean + /-SEM of N = 3, n = 3. D. Percent adhesion of STM WT, STM WT/pQE60: *csrA*, STM Δ*pflB* and STM Δ*pflB*/pQE60: *csrA* in Caco-2 cell line. Data is represented as Mean + /-SEM of N = 3, n = 3. E. TEM assisted visualization of the flagellar structures in STM WT, STM WT/pQE60: *csrA*, STM Δ*pflB*, STM Δ*pflB*/pQE60: *csrA.* Red arrows denote flagellar structures. Data is representative of N = 2, n = 10. F. Raw ITC isotherm between CsrA and sodium formate. G. Fitted ITC isotherm between CsrA and sodium formate. (Unpaired two-tailed Student’s t-test for column graphs, Two-way ANOVA for grouped data, Mann-Whitney U-test for animal experiment data (*** p < 0.0001, *** p < 0.001, ** p < 0.01, * p < 0.05)).

To examine how formate influences the regulation of the flagellar operon, we measured the transcript levels of several genes across its different classes, including class I (*flhD*), class II (*flgA*, *fliA*), and class III (*flgK*, *motA*) (Fig 4A) [42–50]. Our analysis revealed that in the formate-deficient *pflB* knockout strain, inhibition of the flagellar operon occurs at the level of class I genes or their regulatory genes. The transcription of the *flhDC* promoter is governed by multiple factors, such as OmpR, Crp, HdfR, H-NS, QseBC, FliA, and LrhA, among others [44,51–53]. One of the most well-characterized mechanisms of post-transcriptional regulation of *flhDC* involves the Csr system, which comprises the RNA-binding protein CsrA and the small regulatory RNAs (sRNAs) *csrB* and *csrC* [54].

CsrA, a 61-amino-acid protein, binds to the mRNA of target genes, modulating their translation by either inhibiting or enhancing it [55]. Specifically, CsrA binds to the *flhD* transcript, stabilizing it by preventing RNase E-mediated cleavage at the 5′ end [56]. The activity of CsrA is counteracted by the small regulatory RNAs *csrB* and *csrC*, where a single *csrB* RNA can sequester up to 18 CsrA molecules, and a single *csrC* RNA can bind up to 9 CsrA molecules [57–59]. In *S.* Typhimurium, CsrA has been implicated in regulating motility, virulence, carbon storage, and secondary metabolism [60,61]. The transcription of *csrB* and *csrC* is controlled by the BarA/SirA two-component systems (TCS), which are orthologues in *E. coli* and *S.* Typhimurium [62–65].

We observed an increase in *csrB* transcript levels in STM Δ*pflB*, which decreased in STM *ΔpflB* + F and upon plasmid- mediated complementation of the gene (Fig 4B). We also observed a reduction in *csrA* transcript levels in STM Δ*pflB,* which was recovered upon incubation in formate supplemented LB or LB with acidic pH (pH 6) (S8B Fig). Since *csrB* directly binds to CsrA and inhibits its activity, we decided to clone *csrA* into the extragenous plasmid pQE60 and transform this recombinant plasmid into STM Δ*pflB*. Overexpressing *csrA* through IPTG induction partially restored the reduced expression of *fliC* in STM Δ*pflB* (Fig 4C). CsrA is known to bind and stabilize the *flhD* transcript. However, we speculate that an excess of CsrA molecules in the cell could potentially stabilize *flhD* to the point of inhibiting its translation. This may explain why *csrA* overexpression in STM WT led to a decrease in *fliC* expression. Overexpression of *csrA* mediated downregulation of motility has been demonstrated in *Bacillus subtilis* and *Clostridium difficile* as well [66,67]. The increase in *fliC* transcript levels was also associated with a higher adhesion percentage for STM Δ*pflB*/p: *csrA* compared to STM Δ*pflB* (Fig 4D). The increase in flagellar numbers upon *csrA* overexpression in STM Δ*pflB* was visualized using TEM (Fig 4E).

We wanted to understand if formate can directly bind to CsrA and modulate its activity. Hence, we purified the CsrA protein using Ni^2+-^NTA and size exclusion chromatography (SEC). The protein was purified as dimers on an SEC column (S9A,S9B Fig). Circular dichroism (CD) spectra of CsrA showed that the protein consisted of both α-helical and β-sheets (S9C,S9D Fig). The AF3-based modelling also showed that CsrA consists of β-sheet, an α-helix, and loops (S9C,S9D Fig). Taken together, these results show that both the purified proteins were well-folded in solution.

We used isothermal titration calorimetry (ITC) to probe the possible interaction of CsrA with sodium formate. ITC experiments detect the change of heat that is either released or absorbed during a reaction. Analysis of the ITC isotherms gives information on the binding’s dissociation constant (KD) along with other thermodynamic parameters. For formate titrations against CsrA protein, we did not observe significant heat change (**Fig 4F**,**4G**). The minimum heat changes observed at each injection were constant and did not saturate with increasing ligand concentration during the titration, suggesting no interaction between the proteins and the ligand. Therefore, we can conclude that CsrA does not interact directly with the sodium formate under tested experimental conditions, reducing the possibility of such an interaction inside the bacterial cell as well.

### The extracytoplasmic sigma factor RpoE enhances the expression of *csrB*, which in turn limits the expression of *fliC*

In *E. coli*, RpoE (σ^E^) serves as an extracytoplasmic stress sigma factor that is critical for cell viability and for regulating the response to membrane stress [68]. The membrane-bound anti-sigma factor RseA binds to σ^E^, aided by the periplasmic protein RseB [69,70]. Under normal conditions, without envelope stress, RseA binding prevents σ^E^ from associating with RNA polymerase [70–73]. During envelope stress, the proteolytically unstable RseA is rapidly degraded, freeing σ^E^ to perform its downstream functions. These include activating genes responsible for the biogenesis, transport, and assembly of lipopolysaccharides (LPS), phospholipids, and outer membrane proteins (OMPs), as well as proteases and chaperones that help maintain or repair outer membrane (OM) integrity [70].

**Fig 5:**
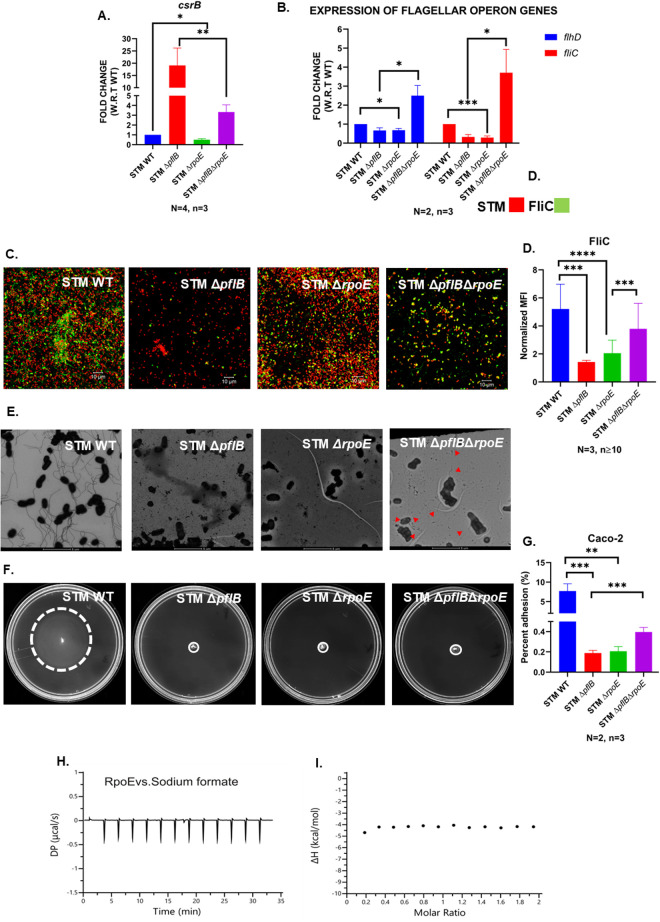
The extra cytoplasmic sigma factor RpoE increases the expression of *csrB* and therefore restricts the expression of *fliC.* A. RT-qPCR mediated expression profile of the sRNA *csrB* in the logarithmic cultures of STM WT, STM Δ*pflB*, STM Δ*rpoE*, STM Δ*pflB*Δ*rpoE.* Data is represented as Mean + /-SEM of N = 4, n = 3. B. RT-qPCR mediated expression profile of the flagellar operon genes *flhD* and *fliC* in the logarithmic cultures of STM WT, STM Δ*pflB*, STM Δ*rpoE*, STM Δ*pflB*Δ*rpoE*. Data is represented as Mean + /-SEM of N = 2, n = 3. C. Confocal microscopy assisted visualization of the flagellar structures in STM WT, STM Δ*pflB*, STM Δ*rpoE*, STM Δ*pflB*Δ*rpoE*. Images are representative of N = 3, n ≥ 10. D. Normalized MFI, i.e.,., MFI of Secondary antibody for Rabbit generated Anti-fli antibody/ MFI for bacterial plasmid mediated mCherry in confocal microscopy assisted visualization of the flagellar structures in STM WT, STM Δ*pflB*, STM Δ*rpoE*, STM Δ*pflB*Δ*rpoE*. Data is representative of N = 3, n ≥ 10 and is represented as mean + /-SD. E. TEM assisted visualization of the flagellar structures in STM WT, STM Δ*pflB*, STM Δ*rpoE*, STM Δ*pflB*Δ*rpoE.* Red arrows denote flagellar structures. Data is representative of N = 2, n = 10. F. Swim motility of STM WT, STM Δ*pflB*, STM Δ*rpoE*, STM Δ*pflB*Δ*rpoE* on 0.25% Agar containing LB plates. G. CFU based percent adhesion of STM WT, STM *Δ*pflB**, STM *Δ*rpoE**, STM *Δ*pflB*Δ*rpoE** in Caco-2 cell line. Data is represented as Mean + /-SEM of N = 2, n = 3. H. Raw ITC isotherm between RpoE and sodium formate. I. Fitted ITC isotherm between CsrA and sodium formate. (Unpaired two-tailed Student’s t-test for column graphs, Two-way ANOVA for grouped data, Mann-Whitney U-test for animal experiment data (**** p < 0.0001, *** p < 0.001, ** p < 0.01, * p < 0.05)).

Recent research has shown that σ^E^ also indirectly enhances the transcription of *csrB* and *csrC* sRNAs via σ^70^ promoters [74,75]. Although these findings were initially established in *E. coli*, deletion of *rpoE* in STM resulted in a reduction in *csrB* transcript levels (~0.588), suggesting that a similar regulatory mechanism may be conserved in STM (Fig 5A).

Our previous data demonstrated that formate produced by PflB helps maintain the ipH of the cell. Consequently, deletion of the *pflB* gene disrupts ipH balance, leading to membrane depolarization. We hypothesized that this membrane stress in STM Δ*pflB* could cause RseA degradation, thereby freeing σ^E^ to promote *csrB* transcription. Our results showed that the elevated *csrB* expression observed in STM Δ*pflB* was significantly reduced in STM Δ*pflB*Δ*rpoE* (Fig 5A). Additionally, the suppressed expression of *flhD* and *fliC* in STM Δ*pflB* was restored in STM Δ*pflB*Δ*rpoE* (Fig 5B). Confocal microscopy and TEM provided further confirmation of this finding (Fig 5C–5E). However, we did not observe any recovery in the swim halo upon deletion of *rpoE* from STM Δ*pflB*, which shows that the recovery in translational level and protein level is not getting reflected in the motility of the attenuated strain (Fig 5F). STM Δ*pflB*Δ*rpoE* exhibited enhanced adhesion to Caco-2 cells compared to STM Δ*pflB*, as demonstrated by both CFU-based adhesion assays and confocal microscopy (Figs 5G,S4A). This improved adhesion might be due to the enhanced hydrophobicity due to increased FliC protein in STM Δ*pflB*Δ*rpoE* compared to STM Δ*pflB* [31]. These results suggest that the reduction in flagella synthesis in STM *ΔpflB* could be mediated by the extra cytoplasmic stress factor σ^E^.

To determine whether formate can also exert its effects by directly binding to the RpoE protein, we purified RpoE following the same approach used for CsrA, utilizing Ni^2+^ -NTA affinity chromatography and size-exclusion chromatography (SEC). On the SEC column, RpoE eluted as monomers (S9E,S9F Fig). CD spectra indicated that RpoE is predominantly α-helical, a structural feature also supported by AF3-based modelling (S9G,S9H Fig). These results collectively confirm that RpoE is properly folded in solution.

During sodium formate titration experiments using ITC, we observed no significant heat changes (Fig 5H,5I). The minimal heat detected at each injection remained consistent and did not reach saturation with increasing ligand concentrations, indicating an absence of interaction between RpoE and formate. Thus, our findings suggest that RpoE does not directly bind sodium formate under the tested conditions, making such an interaction within the cell unlikely.

### Formate regulates the balance between flagellar expression and SPI-1 gene expression in the intestine of C57BL/6 mice

**Fig 6:**
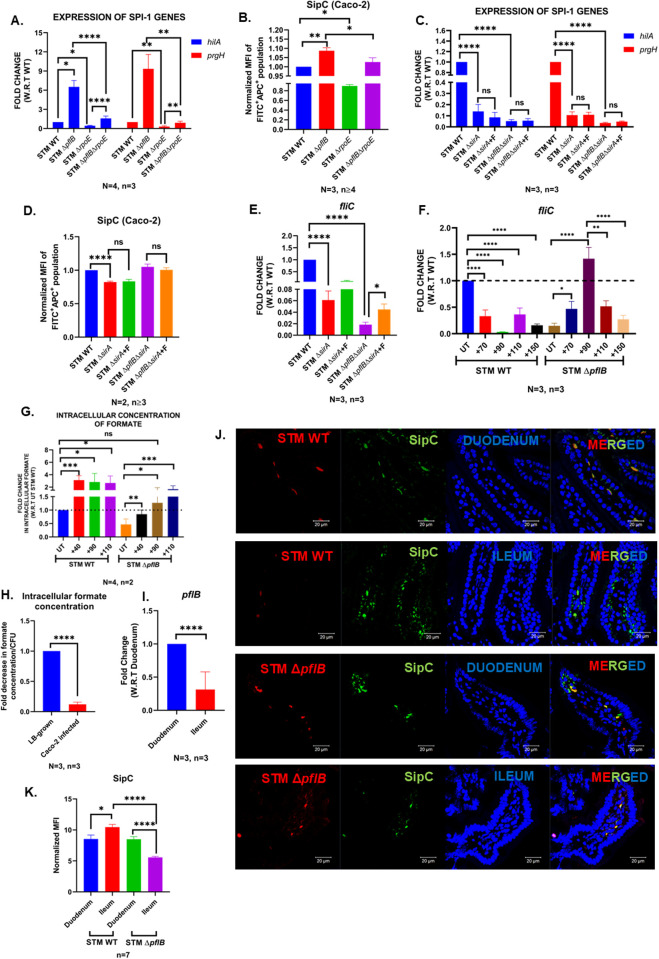
PflB maintains the switch between the flagellar expression and SPI-1 gene expression in intestine of the C57BL/6. A. RT-qPCR mediated expression profile of the SPI-1 genes *hilA* and *prgH* in the logarithmic cultures of STM WT, STM Δ*pflB*, STM Δ*rpoE*, STM Δ*pflB*Δ*rpoE.* Data is represented as Mean + /-SEM of N = 4, n = 3. B. Flow cytometry based normalized MFI of APC (reflecting SipC expression) in FITC positive (bacteria-infected) Caco-2 cells upon infection with STM WT, STM Δ*pflB*, STM Δ*rpoE*, STM Δ*pflB*Δ*rpoE*. Data is represented as Mean + /-SEM of N = 3, n ≥ 4. C. RT-qPCR mediated expression profile of the SPI-1 genes *hilA* and *prgH* in the logarithmic cultures of STM WT, STM Δ*pflB*, STM Δ*sirA*, STM Δ*pflB*Δ*sirA.* Data is represented as Mean + /-SEM of N = 3, n = 3. D. Normalized APC MFI (indicating SipC expression) in FITC-positive (bacteria-infected) Caco-2 cells, measured by flow cytometry after infection with STM WT, STM Δ*pflB*, STM Δ*rpoE*, and STM Δ*pflB*Δ*rpoE*. Data is shown as mean ± SEM of N = 3, n ≥ 4. E. RT-qPCR mediated expression profile of *fliC* gene in the logarithmic cultures of STM WT, STM Δ*sirA*, STM Δ*pflB*ΔsirA (+/-formate)*.* Data is represented as Mean + /-SEM of N = 3, n = 3. F. RT-qPCR mediated expression profile of *fliC* gene in the logarithmic cultures of STM WT and STM Δ*pflB* supplemented with 70, 90, 110, 150 mM of sodium formate. Data is represented as Mean + /-SEM of N = 3, n = 3. G. GC-MS mediated quantification of relative intracellular formate in STM WT, STM Δ*pflB*, supplemented with 40, 90, 110 mM Sodium formate. Data is represented as Mean + /-SEM of N = 4, n = 2 H. Intracellular bacterial formate levels at 30 minutes post-infection in Caco-2 cells, compared to corresponding LB-grown cultures. Data are presented as mean ± SEM from N = 3, n = 3. I. RT-qPCR mediated expression profile of *pflB* gene in the duodenum and ileum of the intestine of C57BL/6 mice*.* Data is represented as Mean + /-SEM of N = 3, n = 3. J. Confocal microscopy assisted visualization of the SPI-1 effector SipC protein in the duodenum and ileum of mice infected with STM WT and STM Δ*pflB*. Images are representative of n = 7. J. Normalized MFI ratio of SipC to bacterial mCherry following confocal microscopy-based visualization of the SPI-1 effector SipC in the duodenum and ileum of mice infected with STM WT or STM Δ*pflB*. Data is represented as mean + /-SD of n = 7. (Unpaired two-tailed Student’s t-test for column graphs, Two-way ANOVA for grouped data, Mann-Whitney U-test for animal experiment data (**** p < 0.0001, *** p < 0.001, ** p < 0.01, * p < 0.05)).

Our previous data showed that STM Δ*pflB* exhibited a higher abundance of the *prgH* transcript. To determine if this increased expression of the SPI-1 gene was controlled by the same regulatory loop, we analysed the transcript levels of the SPI-1 genes *hilA* and *prgH*. We found that the elevated expression of *hilA* and *prgH* in STM Δ*pflB* was reduced in STM Δ*pflB*Δ*rpoE* (Fig 6A). This was accompanied by a reduction in the elevated SipC levels in Caco-2 cells (Figs 6B,S3B). This confirmed that intracellular formate regulates the shift from flagellar expression to SPI-1 gene expression.

Our previous results showed that formate supplementation enhances *prgH* expression in both STM WT and STM Δ*pflB* (Fig 2A). Flagellar motility and epithelial cell invasion are regulated by the BarA/SirA two-component system (TCS) [64]. As expected, STM Δ*sirA* exhibited low levels of *hilA* and *prgH* expression, and formate supplementation in STM Δ*sirA* did not significantly alter the expression of these genes. Additionally, deletion of *sirA* in STM Δ*pflB* eliminated any significant upregulation of *hilA* and *prgH* in response to formate supplementation (Fig 6C). A similar trend was observed for SipC secretion in Caco-2 cell as well (Fig 6D). This indicates that extracellular formate influences the virulence and pathogenicity of *Salmonella* via the BarA/SirA TCS.

Interestingly, we observed that STM Δ*pflB*Δ*sirA* continued to show recovery in *fliC* transcript levels under formate supplementation. This suggests that intracellular formate regulates *Salmonella* pathogenesis independently of the BarA/SirA TCS (Fig 6E). Therefore, when the intracellular formate pool is maintained by the *pflB* gene, extracellular formate modulates SPI-1 gene expression through the BarA/SirA TCS. However, if the intracellular formate pool is disrupted, the resulting membrane stress triggers RpoE-mediated overexpression of *csrB*, which is associated with reduced *fliC* transcript levels and increased *hilA* and *prgH* expression.

As expected, STM Δ*sirA* exhibited low expression levels of *hilA* and *prgH*, and formate supplementation in STM Δ*sirA* did not result in any significant change in the expression of these genes. Furthermore, deletion of *sirA* in STM Δ*pflB* eliminated any significant upregulation of *hilA* and *prgH* in response to formate supplementation (**Fig 6C**). This demonstrates that extracellular formate influences the virulence and pathogenesis of *Salmonella* through the BarA/SirA TCS. Interestingly, we observed that STM Δ*pflB*Δ*sirA* continued to show recovery in *fliC* transcript levels with formate supplementation. This indicates that intracellular formate regulates *Salmonella* pathogenesis independently of the BarA/SirA TCS (**Fig 6E**). Thus, when the intracellular formate pool is maintained by the *pflB* gene, extracellular formate modulates SPI-1 gene expression via the BarA/SirA TCS. However, if the intracellular formate pool is disrupted, the resulting membrane stress triggers RpoE-mediated overexpression of *csrB*, which is associated with reduced *fliC* transcript levels and increased *hilA* and *prgH* expression.

We proceeded to understand whether achieving intracellular formate concentrations of STM Δ*pflB* to similar levels as STM WT and further supplementing it with formate can replicate the behaviour of wild-type strain in presence of formate. We subcultured the stationary phase cultures of STM WT and STM Δ*pflB* and supplemented them higher concentrations of formate (70, 90, 110, 150 mM). We found that there was a substantial decrease in the *fliC* transcript level in STM WT upon supplementation of higher concentrations of formate. STM Δ*pflB* showed a remarkable recovery in the *fliC* transcript level at 90 mM concentration, following which the expression levels declined in the presence of 110 mM and 150 mM formate (Fig 6F). GC-MS mediated quantification of intracellular formate concentration revealed that a concentration of 90 mM was enough to replenish the depleted intracellular formate pool in STM *ΔpflB,* which explains the highest expression of *fliC* at this concentration (Fig 6G). Beyond this, supplementation of formate reduces the expression of *fliC* as seen in Fig 6F. This mimics the scenario of supplementing formate to STM WT with an intact formate pool. This was also visualized under TEM (S10 Fig).

To investigate whether the downregulation of intracellular formate levels is indeed a strategy employed by *Salmonella* during infection, we collected bacterial cells from infected Caco-2 cells 30 minutes post-infection. Using kit-based assays, we quantified intracellular formate and observed a significant decrease in the formate pool of bacteria recovered from infected Caco-2 cells compared to the LB-grown culture used for infection (Fig 6H). Earlier studies have demonstrated that *Salmonella* reduces *pflB* expression as part of its metabolic adaptation during infection. Transcriptomic data indicate a gradual shift from PFL-dependent fermentation to respiration via pyruvate dehydrogenase (PDH), which contributes to NADH production in the early stages of infection [76]. Using *Salmonella*-specific primers, we also observed a steady decline in *pflB* expression from the duodenum to the ileum, the primary site of infection (Figs 6I,S11B,S13 Data). We hypothesized that this downregulation could induce a partial formate deficiency within the bacterial cell, causing membrane depolarization, which in turn activates σ^E^, increases *csrB* abundance, downregulates flagellar operon transcription, and upregulates the SPI-1 gene cascade, ultimately help in bacterial invasion at its target site of ileum.

To test this hypothesis, we collected duodenum and ileum sections from mice infected with STM WT and STM Δ*pflB* and stained them with an anti-SipC antibody to measure levels of the SPI-1 effector protein (Fig 6J). We normalized the fluorescence from the secondary antibody, specific to the mouse anti-SipC antibody, against mCherry fluorescence from bacteria transformed with the pFPV plasmid. In mice infected with STM WT, we found that the normalized MFI increased from the duodenum to the ileum. In contrast, STM Δ*pflB*-infected mice showed a decrease in normalized MFI from the duodenum to the ileum (Fig 6K). To verify SipC secretion in host cells, we performed 3D reconstruction of our histopathology images and observed that the SipC and DAPI signals reside within the same optical plane (S11A Fig). Altogether, these results indicate that the formate pool maintained by the PflB enzyme regulates SPI-1 gene expression, and its depletion may be a strategy used by *Salmonella* Typhimurium to maximize SPI-1 expression, and thereby invasion, in the ileum.

## Discussion

*Salmonella* possesses a network of genes and regulatory factors that manages the balance between its extracellular and intracellular modes of existence [77]. Research on *Salmonella* Typhimurium has demonstrated overlapping regulatory mechanisms between the flagellar secretion system and the SPI-1. The flagellar protein FliZ is known to activate the primary regulator of flagella synthesis, FlhD, through post-translational modification. Kage *et al*. have shown that FliZ can also regulate HilD, a key regulator of SPI-1, at the transcriptional level [78,79]. HilD has been found to bind directly to the promoter region of *flhDC*, upstream of the P5 transcriptional start site, thus enhancing its expression [80]. Other studies indicate that SPI-1 expression can suppress flagellar gene expression, aiding in pathogenicity and virulence of *Salmonella*. A mutation in *fliZ* reduces *hilA* expression by half, while a knockout of *sirA* decreases *hilA* expression tenfold and increases flagella expression a hundredfold [81–84]. Furthermore, recent research by Saleh *et al*. has shown that HilD activation can trigger a SPI-1 dependent induction of the stringent response and significantly reduce the Proton Motive Force (PMF). Although flagellation remains unaffected, HilD activation results in a motility defect in *S.* Typhimurium [85]. Collectively, these studies reveal that *Salmonella* can toggle between flagella synthesis and SPI-1 gene expression to optimize adhesion and the delivery of SPI-1 effectors. There are several potential benefits to downregulating flagella while upregulating SPI-1 genes. First, reducing flagella expression prevents the bacteria from moving away from a target cell primed for invasion. Second, because the flagellar apparatus is immunogenic, downregulating it may help the bacteria evade the host immune response [86–88]. Lastly, simultaneous production of both systems could lead to interference, potentially causing the improper secretion of flagellin and SPI-1 effectors [89].

In our study, we knocked out the gene encoding the pyruvate-formate lyase enzyme, effectively depleting the cell of its native formate pool. Although the knockout strain displayed increased expression of the SPI-1 gene *prgH*, it showed reduced transcript levels of *fliC*. This was reflected in decreased invasion and adhesion capabilities in a human epithelial cell line. These deficiencies were partially restored with the addition of 40 mM formate. Previous studies have reported that *Salmonella* mutants lacking flagella exhibit increased net growth in mouse macrophages. Consistent with these findings, we observed that STM Δ*pflB*, which is also flagella-deficient, showed hyperproliferation in the murine macrophage cell line RAW 264.7 [23]. In a mouse model, we observed that infection with STM Δ*pflB* resulted in a reduced organ burden in the liver and spleen at 5 dpi following oral gavage, despite its adhesion deficiency similar to STM Δ*fliC*, which showed organ burdens comparable to STM WT at the same time point. These findings suggest that STM Δ*pflB* lacks key factors like flagella amongst others required for intestinal colonization.

We found that the STM Δ*pflB* mutant could not maintain its internal pH as well as the wild type, which led to membrane depolarization. We initially hypothesized that this membrane stress activated the sigma factor RpoE, increasing small RNA *csrB* expression. Since *csrB* binds to and blocks the CsrA protein and CsrA is responsible for maintaining the stability of *flhD* (master regulator of flagellar synthesis), we suspected that higher *csrB* levels reduced *fliC* expression. When we overexpressed *csrA* using an external plasmid, *fliC* expression partially recovered, supporting our idea. Deleting *rpoE* in the STM Δ*pflB* strain also restored flagella formation, further confirming that RpoE responds to membrane stress and affects downstream processes.

In all our experiments, we noticed that adding formate reduced flagellar expression in the STM WT. Interestingly, extracellular formate supplementation did not change the internal pH or membrane potential of the WT, suggesting that external formate affects *Salmonella* differently than formate made inside the cell. Huang *et al*. had showed that external formate boosts invasion, but this effect did not depend on the BarA/SirA system or *csrB*, which is normally regulated by SirA. In our study, we found that formate-supplemented STM WT had higher SPI-1 gene expression [90]. Unlike earlier reports, we also observed increased *csrB* levels in the WT with formate, which may explain the drop in *fliC* expression [91]. This suggests that formate is an important metabolite for *Salmonella*, and its effect depends on its concentration—either acting through the two-component system or by affecting internal pH. We also found that adding formate to the STM Δ*pflB* strain restored its internal formate pool and made it behave like the wild type, showing reduced *fliC* expression when formate was present.

*Salmonella* Typhimurium has been extensively studied for its ability to adapt its metabolism to conditions within the host. Previous research has shown that spatially distinct *Salmonella* populations in the mucous layer and intestinal lumen differ in their energy metabolism. Transcript levels of pyruvate-formate lyase and formate dehydrogenases were found to be higher in the mucous layer than in the lumen population [15]. This formate metabolism provided a fitness advantage in the mucous layer. Previous research using Low Complexity Microbiota (LCM) models has shown that *Salmonella* downregulates *pflB* as part of its metabolic adjustment during infection. Transcriptomic data indicate a progressive shift from PFL-dependent fermentation toward pyruvate dehydrogenase (PDH)-based respiration, associated with NADH-driven energy production in the early stages of infection [76]. In our study, we also observed that *pflB* transcription was highest in the duodenum and gradually decreased in the ileum, the primary site of *Salmonella* infection. This suggests that *Salmonella* modulates *pflB* expression in response to local gut microenvironment signals. We propose a model in which *Salmonella* downregulates the *pflB* gene upon reaching its infection site, disrupting its intracellular formate pool. This can result in partial membrane depolarization, which downregulates the flagellar apparatus and promotes invasion into epithelial cells through upregulated expression of the SPI-1 system. Although it is difficult to reconcile how a regulatory circuit would prioritize SPI-1 expression during a depolarized membrane state that could impair T3SS-1 functionality, we have seen from our cell line data that SPI-1 effector secretion was enhanced, showing that SPI-1 assembly and functionality is intact. There is a possibility that the residual membrane potential is being repurposed to drive SPI-1 secretion upon host cell contact. *In vitro*, approximately 40% of STM Δ*pflB* cells exhibited membrane depolarization, consistent with a full knockout. In contrast, *pflB* expression *in vivo* was reduced by about 20% in *Salmonella*, indicating a less pronounced depolarization. Both *pflB* downregulation and membrane depolarization in the intestine are likely transient, with a shift to regularity after impacting the downstream processes. Moreover, our findings are consistent with Saleh *et al.*, who showed that HilD-driven SPI-1 activation causes membrane depolarization, reducing motility while promoting effector secretion [85].Overall, our study contributes to understanding how intracellular metabolism in *Salmonella* influences its ability to adhere to and invade non-phagocytic intestinal epithelial cells, and it highlights the potential for uncovering additional host-pathogen metabolic interactions in future research.

## Materials and methods

### Ethics statement

All the animal experiments have been approved by the Institutional Animal Ethics Clearance Committee (IAEC), Indian Institute of Science, Bangalore. The registration number is 48/1999/CPCSEA. The guidelines formulated by the Committee for the Purpose of Control and Supervision of Experiments on Animals (CPCSEA) were strictly followed. The ethical clearance number for this study is CAF/Ethics/853/2021.

### Bacterial strains and plasmids

The wild-type *Salmonella enterica* subspecies *enterica* serovar Typhimurium strain 14028S used in this study was generously provided by Professor Michael Hensel from the Max von Pettenkofer Institute for Hygiene and Medical Microbiology, Germany. Knockout and complement strains were generated specifically for this study. Bacterial strains were stored as glycerol stocks at -80°C and revived on fresh Luria-Bertani (LB) agar plates (HiMedia) with appropriate antibiotics as needed (50 µg/ml Kanamycin, 50 µg/ml Ampicillin, 25 µg/ml Chloramphenicol). A single colony was inoculated into a fresh LB tube with antibiotics when required and incubated at 37°C with shaking (170 rpm) to obtain an overnight culture. Strains containing the pKD46 plasmid were incubated overnight at 30°C with shaking (170 rpm). A complete list of bacterial strains and plasmids used in this study is provided in Table 1.

**Table 1:** List of strains.

Strains/ plasmids	Characteristics	Source/Information
*Salmonella enterica* subspecies *enterica* serovar Typhimurium strain 14028S	Wild type (WT)	Gift from Prof. M. Hensel
*S*. Typhimurium Δ*focA*	Chl^R^	This study
*S*. Typhimurium Δ*pflB*	Kan^R^	This study
*S*. Typhimurium Δ*pflB*/pQE60	Kan^R^, Amp^R^	This study
*S*. Typhimurium Δ*pflB*/pQE60: *pflB*	Kan^R^, Amp^R^	This study
*S*. Typhimurium Δ*focA*Δ*pflB*	Kan^R^, Chl^R^	This study
*S*. Typhimurium Δ*fliC*	Kan^R^	Laboratory stock
*S*. Typhimurium/pQE60: *csrA*	Amp^R^	This study
*S*. Typhimurium Δ*pflB*/pQE60: *csrA*	Kan^R^, Amp^R^	This study
*S*. Typhimurium Δ*rpoE*	Chl^R^	This study
*S*. Typhimurium Δ*pflB*Δ*rpoE*	Kan^R^, Chl^R^	This study
*S*. Typhimurium Δ*sirA*	Chl^R^	This study
*S*. Typhimurium Δ*pflB*Δ*sirA*	Kan^R^, Chl^R^	This study
*S*. Typhimurium Δ*sipC*	Chl^R^	This study
pKD4	Plasmid with FRT flanked Kanamycin-resistant cassette	
pKD3	Plasmid with FRT flanked Chloramphenicol-resistant cassette	
pKD46	Plasmid expressing λ-red recombinase system, Amp^R^	
pQE60 vector	Low copy number plasmid, Amp^R^	Laboratory stock
pHuji		Addgene
pFPV	Low copy plasmid encoding mCherry protein, Amp^R^	Laboratory stock

### Construction of the knockout strains of *Salmonella*

The knockout strains of *Salmonella* were generated using the one-step chromosomal gene inactivation technique developed by Datsenko and Wanner [19]. In brief, STM WT was transformed with the pKD46 plasmid, which expresses the λ-Red recombinase system under the control of an arabinose-inducible promoter. Transformed cells were selected on LB plates containing 50 µg/ml ampicillin. To generate the knockout strains, a single colony of STM WT/pKD46 was inoculated into fresh LB medium with 50 µg/ml ampicillin and 50 mM arabinose, then incubated overnight at 30°C with shaking (170 rpm). The overnight culture was then subcultured into fresh LB medium and incubated under the same conditions for 2.5 hours to reach an OD_600_ of 0.35-0.4. Electrocompetent STM WT/pKD46 cells were prepared by washing the bacterial pellet with chilled, autoclaved milliQ water and 10% glycerol. Kanamycin (Kan^R^, 1.5 kb) and chloramphenicol (Chl^R^, 1.1 kb) resistance cassettes were amplified from pKD4 and pKD3 plasmids, respectively, using knockout-specific primers. The amplified resistance cassettes were then electroporated into STM WT/pKD46, and transformed cells were selected on LB agar plates with the appropriate antibiotics (kanamycin or chloramphenicol). Knockout colonies were confirmed using gene-specific intragenic primers, knockout confirmatory primers, and antibiotic-cassette-specific primers.

For generating double-knockout strains, the one-step chromosomal gene inactivation method was slightly modified. Briefly, STM Δ*pflB* was transformed with the pKD46 plasmid. An overnight culture of the transformed strain was subcultured into fresh LB medium supplemented with 50 mM arabinose and 50 µg/ml Ampicillin, then incubated at 30°C with shaking (170 rpm) for 2.5 hours until it reached an OD600 of 0.3-0.4. The chloramphenicol resistance cassette (1.1 kb), flanked by regions homologous to *focA*, was electroporated into electrocompetent STM Δ*pflB*/pKD46 cells to generate STM Δ*focA*Δ*pflB*. The double-knockout strains were selected on LB agar plates containing kanamycin (50 µg/ml) and chloramphenicol (25 µg/ml). These double-knockout colonies were further confirmed with knockout confirmatory and gene-specific intragenic primers. The primers used for generating knockouts are listed in Table 2.

**Table 2:** List of primers.

PRIMER NAME	SEQUENCE (5’-3’)
16S rRNA RT FP	GTGAGGTAACGGCTCACCAA
16S rRNA RT RP	TAACCGCAACACCTTCCTCC
*hilA* RT FP	GCCGGTGACCATTACGAAGA
*hilA* RT RP	AAGAGAGAAGCGGGTTGGTG
*prgH* RT FP	CGTTTTCAGGTGTTGCCAGG
*prgH* RT RP	TACGCGGCTCATCGAAATGA
*flhD* RT FP	CGCCTCGGTATCAACGAAGA
*flhD* RT RP	CACTTCATTGAGCAGACGCG
*fliC* RT FP	CTAAACAAACTGGGTGGCGC
*fliC* RT RP	GCACCCAGGTCAGAACGTAA
*flgA* RT FP	ATTATGTCGCCGTTGCC
*flgA* RT RP	TGACCTGTACTCGTTGC
*flgK* RT FP	AACCACCGATCAGTATC
*flgK* RT RP	TCGTCAGGTTATAGGTG
*fliA* RT FP	GCTGGATGAATTACGCA
*fliA* RT RP	CCACTCATCGTAAGAGA
*motA* RT FP	TTGCTGGCGTTGCTCTA
*motA* RT RP	GATGATCAGGCGCAGAT
*csrB* RT FP	CGTACAACGAAGCGAACGTC
*csrB* RT RP	TGTGACCTTACGGCCTGTTC
*focA* KO FP	ACACCGTAATTGCATAAAAGCCATGCGACTTACGGGCCTACATATGAATATCCTCCTTAG
*focA* KO RP	ATGCTCGTTACCACGCAGGTAAATGACCCAGTATGTCAACGTGTAGGCTGGAGCTGCTTC
*pflB* KO FP	ATGTCCGAGCTTAATGAAAAGTTAGCCACAGCCTGGGAACATATGAATATCCTCCTTAG
*pflB* KO RP	TACATGGTCTGCGTGAAGGTACGAGTAATAACGTCCTGCTGTGTAGGCTGGAGCTGCTTC
*focA* RT FP	CGGAAAGCCACATCAGTAAC
*focA* RT RP	ATCGGGGGTATCTGTTTCTC
*pflB* RT FP	ATAACGTCCTGCTGCTGTTC
*pflB* RT RP	ATGGTTACTTCCACCACGAA
*pflB* cloning FP	CGCGGATCCATGTCCGAGCTTAATGA
*pflB* cloning RP	CCCAAGCTTCAGTCAAACCCATTACA
*rpoE* KO FP	GGTTTGGGGAGACATTACCTCGGATGAGCGAGCAGTTACATATGAATATCCTCCTTAG
*rpoE* KO RP	CCTAATACCTTTTCCAGTATCCCGCTATCGTCAACGGTGTAGGCTGGAGCTGCTTC
*sirA* KO FP	CTATCAGTAGCGTTATCCCTATTCTGGAGATATTCCTTTGCATATGAATATCCTCCTTAG
*sirA* KO RP	ATACGATAGCGATAGCTGTTCACCGTTTTAGGACTGAGATGTGTAGGCTGGAGCTCTTC
*csrA* cloning FP	CGCGGATCCATGCTGATTCTGACTCG
*csrA* cloning RP	CCCAAGCTTTTAGTAACTGGACTGCTGG
*sipC* KO FP	GTATTGGTATTAGCAGCAGTAAAGTCAGTGACCTGGGGTTCATATGAATATCCTCCTTAG
*sipC* KO RP	CAGCATTTCCTGAATCAGGCTGGTCGATTTACGTGAACTTGTGTAGGCTGGAGCTGCTTC
*sipC* KO confirmatory FP	TGCCATGGATCAGATTCAGC
*sipC* KO confirmatory RP	TACCGCGATGTTCTGTGGTA
STM specific *pflB* RT FP	GCAAACCTGGCGAAAACCAT
STM specific *pflB* RT FP	TCGGATTTCGGACCAACC

### Construction of complemented and overexpression strains of *Salmonella*

The *pflB* and *csrA* genes were amplified from the genomic DNA of wild-type *Salmonella* using gene-specific cloning primers. Both the colony PCR products and the empty pQE60 cloning vector were digested with BamHI-HF and HindIII-HF (NEB) at 37°C for 1 hour. After double digestion, the insert and vector were purified and ligated using T4 DNA Ligase (NEB) at 16°C overnight. The ligation product was then transformed into *Escherichia coli* TG1 using the heat shock method. Successful cloning was confirmed by double digestion of the recombinant plasmid. The confirmed recombinant plasmid was subsequently transformed into *Salmonella* strains to create complemented and overexpression strains. Primers used for generating complement strains are listed in Table 2.

### RNA Isolation and RT-qPCR

Logarithmic-phase cultures of bacterial cells were centrifuged at 6,000 rpm at 4°C, and the resulting pellet was resuspended in TRIzol reagent. The suspension was stored at -80°C until further use. RNA was isolated from the lysed supernatants using the chloroform-isopropanol method. The RNA pellet was washed with 70% ethanol and resuspended in 30 µl of DEPC-treated milliQ water. RNA concentration was measured with a NanoDrop spectrophotometer (Thermo Fisher Scientific), and quality was assessed on a 2% agarose gel. Following this, 2 µg of RNA was DNase treated using DNase TURBO (Thermo Fisher Scientific) at 37°C for 30 minutes. DNase was inactivated by adding 5 mM Na₂EDTA (Thermo Fisher Scientific) and incubating at 65°C for 10 minutes. cDNA synthesis was carried out using the PrimeScript RT reagent kit (Takara, CAT RR037A) according to the manufacturer’s instructions. Quantitative real-time PCR (qRT-PCR) was performed with a TB Green RT-qPCR kit (Takara) on the Biorad and QuantStudio 5 Real-Time PCR system. Intragenic primers were used to examine gene expression, and expression levels were normalized to 16S rRNA. Primer sequences are listed in Table 2.

### Eukaryotic cell lines and growth conditions

The Caco-2 and RAW 264.7 cell lines were cultured in Dulbecco’s Modified Eagle’s Medium (DMEM, Sigma-Aldrich) supplemented with 10% Fetal Calf Serum (FCS, Gibco), at 37°C with 5% CO₂.

### Gentamicin protection assay and intracellular cell survival assay

Caco-2 and RAW 264.7 cells were seeded into tissue culture-treated 24-well plates. For infection of the epithelial Caco-2 cells, overnight cultures of STM WT, STM Δ*pflB*, and STM Δ*pflB*/pQE60: *pflB*, were subcultured in fresh LB medium, with or without supplementation of 40 mM sodium formate (Sigma). For infection of the macrophage cell line RAW 264.7, the overnight cultures were used directly. Both cell types were infected at a multiplicity of infection (MOI) of 10. The plates were centrifuged at 1000 rpm for 10 minutes, after which the cells were incubated at 37°C with 5% CO₂ for 25 minutes. Following incubation, cells were washed with 1X phosphate-buffered saline (PBS) to remove extracellular, non-adherent, and loosely adherent bacteria, and fresh media containing 100 µg/ml gentamicin was added. After a 1-hour incubation, cells were washed again with 1X PBS and exposed to 25 µg/ml gentamicin until lysis. Cell lysis was performed at 2 hours and 16 hours post-infection using 0.1% Triton X-100, and the lysates were plated onto *Salmonella*-*Shigella* (SS) agar alongside the pre-inoculum. The percent invasion and fold proliferation were determined using the following formulas:


\textrm{Percent invasion/phagocytosis =[C.F.U at 2h/C.F.U of Pre−Inoculum]*100}

{Fold proliferation = [C.F.U at 16h/C.F.U of 2h]}

### Intracellular bacteria visualization using confocal microscopy

Caco-2 cells seeded on coverslips were infected with STM WT/pFPV: mCherry, STM Δ*pflB*/pFPV: mCherry (with or without formate treatment), STM Δ*rpoE*/pFPV: mCherry, and STM Δ*pflB*Δ*rpoE*/pFPV:mCherry at an MOI of 10, following previously described procedures. After treatment with 100 µg/ml and 25 µg/ml gentamicin for one hour each, the medium was discarded, and the cells were washed once with 1X PBS. The cells were then fixed using 3.5% paraformaldehyde for 10 minutes. Subsequently, coverslips were incubated with rabbit anti-human LAMP-1 antibody (CST, 9091S) at a 1:300 dilution in BSA-saponin solution for 3 hours at room temperature. After removing the primary antibody and washing with 1X PBS, the samples were incubated with an anti-rabbit Alexa Fluor 488-conjugated secondary antibody for 45 minutes at room temperature. Following secondary antibody removal and PBS washing, the coverslips were mounted on clear glass slides and sealed with transparent nail polish. Imaging was conducted using a Zeiss 880 microscope (63X oil immersion objective), and images were analysed with Zeiss ZEN Black 2012 software.

### Adhesion assay by CFU enumeration

The adhesion assay was performed by modifying the previously reported protocol [92]. Overnight cultures (12 hours old) of STM WT, STM Δ*pflB*, STM Δ*pflB*/pQE60: *pflB*, STM WT/ pQE60: *csrA*, STM Δ*pflB*/pQE60: *csrA, STM* Δ*rpoE, STM* Δ*pflB* Δ*rpoE,* and STM Δ*fliC* were subcultured into fresh LB medium, with or without supplementation of 40 mM sodium formate (Sigma). The logarithmic phase of these bacterial strains was used to infect the cells at a MOI of 10. The plates were centrifuged at 1000 rpm for 10 minutes, followed by incubation at 37°C with 5% CO₂ for 10 minutes. To remove extracellular, unadhered, and loosely adhered bacteria, the cells were washed twice with 1X PBS. Subsequently, the cells were lysed with 0.1% Triton X-100, and the lysates were plated on *Salmonella-Shigella* (SS) agar alongside the pre-inoculum. The percent adhesion was determined using the following formula:


{Percent adhesion=[C.F.U at 10 mins Post Infection/C.F.U of Pre−Inoculum]*100}

### Adhesion assay by confocal microscopy

The adhesion assay was conducted by modifying a previous protocol [93]. 12-hour overnight cultures of STM WT, STM Δ*pflB*, STM Δ*rpoE*, and STM Δ*pflB*Δ*rpoE* were subcultured into fresh LB medium, either with or without the addition of 40 mM sodium formate (Sigma). Bacteria in the logarithmic growth phase were used to infect Caco-2 cells at a MOI of 10. The culture plates were centrifuged at 1000 rpm for 10 minutes, then incubated at 37°C in a 5% CO₂ atmosphere for an additional 10 minutes. To eliminate non-adherent and loosely bound bacteria, the cells were washed twice with 1X PBS. The samples were fixed with 3.5% paraformaldehyde (PFA), which was subsequently removed by washing with 1X PBS. For extracellular bacterial staining, samples were treated with a rabbit anti-*Salmonella* primary antibody (1:200 dilution in 2.5% BSA, Sigma) for 3 hours at room temperature. After washing off the primary antibody with 1X PBS, the samples were incubated with an anti-rabbit secondary antibody (Jackson) for 45 minutes at room temperature. The antibody solution was discarded, and the coverslips were sealed using transparent nail polish. Imaging was performed with a Zeiss 880 microscope (63X oil immersion objective), and image analysis was carried out using Zeiss ZEN Black 2012 software. To enhance clarity, the figure includes z-stacks focused on Caco-2 cells as well as z-stacks highlighting the adhered bacteria.

### Determination of percent of invading bacteria/ adhering bacteria

Log-phase cultures of STM WT, STM Δ*pflB*, and STM Δ*pflB*/pQE60: *pflB* (with or without formate supplementation) were used to infect Caco-2 cells at a MOI of 10. The infection plates were centrifuged at 1000 rpm for 10 minutes, then incubated at 37°C in a 5% CO₂ atmosphere. After 10 minutes of incubation, the medium was removed, and the cells were washed twice with 1X PBS to eliminate extracellular bacteria. Half of the infected Caco-2 wells were lysed with 0.1% Triton X-100, and the lysates were collected to determine colony-forming units (CFU). At 30 minutes post-infection, a 100 µg/ml gentamicin treatment was applied for 1 hour to kill any remaining extracellular bacteria. This was followed by a 25 µg/ml gentamicin treatment for an additional hour. Afterward, the remaining wells were lysed with 0.1% Triton X-100, and lysates were collected for CFU analysis.

The percentage of invading or adhering bacteria was calculated using the following formula:


(\textrm{CFU at 2 hours post−infection/CFU at 10 minutes post−infection)}×\textrm{100}

### Bacterial swim motility

Bacterial swim motility was performed by modifying a protocol from Nair *et al.* [94]. Briefly, 10 µl of logarithmic phase cultures of each strain were spotted on the 0.3% Agar plate, supplemented with 0.5% yeast extract, 1% casein enzyme hydrolysate, 0.5% sodium chloride, and 0.5% glucose. For formate supplementation experiments, 40 mM sodium formate was supplemented directly to the swim agar plates. The plates were incubated at 37˚C incubator and the motility halos were observed and imaged after 6h under Biorad chemidoc.

### TEM

Transmission Electron Microscopy was performed by modifying the protocol by Marathe *et al* [95]. In brief, overnight cultures of STM WT, STM Δ*pflB*, STM WT/*csrA*, STM Δ*pflB*/*csrA*, STM Δ*rpoE*, and STM Δ*pflB*Δ*rpoE* were subcultured (1:100) in fresh LB medium, with or without formate supplementation, and incubated at 37°C for 2.5–3 hours under shaking conditions. The bacterial samples were then washed twice with 1X PBS and resuspended in 50 µl of 1X PBS. A 5 µl aliquot of the suspension was applied to a glow-discharged copper grid and allowed to air-dry. The bacteria were subsequently negatively stained using 1% uranyl acetate. After air-drying, the samples were imaged using Transmission Electron Microscopy (JEM-100 CX II; JEOL).

### Flagella staining by confocal microscopy

Confocal Microscopy was performed by modifying the protocol by Marathe *et al.* [95]. In summary, 12-hour-old stationary-phase cultures of STM WT/pFPV: mCherry, STM Δ*pflB/* pFPV: mCherry, STM Δ*rpoE*/pFPV: mCherry, and STM Δ*pflB*Δ*rpoE*/pFPV: mCherry, with or without formate supplementation, were subcultured (1:100) into fresh LB medium and incubated at 37°C for 2.5–3 hours under shaking conditions until reaching the logarithmic phase. A 100 µl aliquot of the log-phase culture was spotted onto sterile coverslips and air-dried overnight. Once completely dried, the samples were fixed with 3.5% PFA. The PFA was washed off with 1X PBS, and the samples were stained with a rabbit-raised anti-Fli primary antibody (1:100 dilution in 2.5% BSA solution, Difco) for 3 hours at room temperature. After washing off the primary antibody with 1X PBS, the samples were incubated with Alexa-488-conjugated anti-rabbit secondary antibody (Sigma) for 45 minutes at room temperature. The antibody solution was removed, and the coverslips were dried before sealing with transparent nail polish. All images were captured using a Zeiss 880 microscope (63X oil immersion objective) and analyzed with Zeiss ZEN Black 2012 software.

### Gas chromatography-mass spectrometry

GC-MS was performed by modifying the protocol by Hughes *et al.* [96]. Briefly, overnight cultures of STM WT, STM Δ*pflB*, STM *ΔpflB*/pQE60: *pflB*, STM Δ*focA*, STM Δ*focA*Δ*pflB* were subcultured (1:100) into 100 ml of LB Broth. After 2.5-3h of incubation at 37˚C under shaking conditions, 50 ml of the individual cultures were centrifuged at 6,000 rpm for 10 mins. The pellet was washed twice with 1X PBS to remove the remnants of the media. Finally, the pellet was resuspended in 1 ml of 1X PBS, and the suspension was sonicated to lyse the bacterial cells. The cell debris was removed by centrifugation. Before sonication, the CFU in the pellet had been recorded by plating.

Sodium formate (Sigma) was used as an external standard. External standards and biological samples were derivatized before the Mass Spectrometry. 2 M hydrochloric acid was added in a 1:1 ratio to the samples to protonate formate. Liquid-liquid extraction was done which extracted formic acid into ethyl acetate (Sigma-Aldrich). To remove any remaining water, the ethyl acetate extract was passed through a column of anhydrous sodium sulfate (Sigma-Aldrich). The ethyl acetate extract of formic acid was then incubated at 80 ºC for 1 h with the derivatization reagent N, O-Bis(trimethylsilyl)trifluoro acetamide (Sigma Aldrich) with 1% Chlorotrimethylsilane (Fluka). The derivatized samples were then transferred to autosampler vials for analysis by Gas Chromatography Mass Spectrometry (Agilent technologies, 7890A GC, 5975C MS). The injection temperature was set to 200°C and the injection split ratio was 1:40 with an injection volume of 1 µL. The oven temperature was set to start at 40°C for 4 min, with an increase to 120°C at 5°C per min and to 200°C at 25°C per min with a final hold at this temperature for 3 min. Helium carrier gas had a flow rate of 1.2mL/min with constant flow mode. A 30 m × 0.25 mm × 0.40 µm DB-624 column from Agilent was used. The interface temperature was set to 220°C. The electron impact ion source temperature was at 230°C, with an ionization energy of 70 eV and 1235 EM volts. For qualitative experiments, selected ion monitoring (single quadrupole mode) of 103 m/z scan had been performed. The retention time for formate and deuterated formate was 5.9 min. The target and reference (qualifier) ions for formate were m/z = 103 and m/z = 75, respectively. The formate concentration of each sample was interpolated from a standard curve with the known standards. It was normalized with the CFU values of each sample to obtain the intracellular formate concentration/CFU.

To determine the formate transportation into the cell, we subcultured overnight cultures of STM WT, STM *ΔpflB*, STM *ΔfocA*, and STM *ΔfocAΔpflB* into fresh LB tubes. During the subculture, Sodium Formate was supplemented into each tube to maintain a concentration of 1M. Post 2.5-3h of incubation at 37˚C under shaking conditions, the bacterial cells were removed from the media by centrifuging the culture twice at 6,000 rpm for 10 mins. A 2 µm filter was used to remove any other bacterial cells left in the spent media. Following this, a similar protocol as mentioned above was used to quantify the residual formate in the spent media. It was compared to the media where bacterial cells had not been subcultured.

### SipC secretion using flow cytometry

Caco-2 cells were infected with logarithmic phase cultures of STM WT, STM Δ*pflB*, STM Δ*pflB*/pQE60: *pflB* (with or without formate supplementation), STM Δ*rpoE*, STM Δ*pflB*Δ*rpoE*, STM Δ*sirA*, STM Δ*pflB*Δ*sirA*, and STM Δ*sipC* at an MOI of 50. After centrifugation at 1000 rpm for 10 minutes and 30 minutes of infection, extracellular bacteria were removed with PBS washes. Cells were then fixed with 3.5% paraformaldehyde. Intracellular *Salmonella* and SipC were stained using rabbit anti-*Salmonella* and mouse anti-SipC antibodies in 2.5% BSA-Saponin solution, followed by incubation for 3 hours at room temperature. After PBS washes, secondary antibodies—Goat anti-Rabbit Alexa 488 and Goat anti-Mouse Dylight 647 (Jackson)—were added and incubated for 45 minutes. Cells were washed and resuspended in PBS for flow cytometry. Alexa 488 and Dylight 647 signals were detected using FITC and APC filters, respectively. FITC-positive cells were gated, and APC fluorescence was quantified. All flow cytometry was performed and data analysed using a BD FACSVerse (BD Biosciences, USA).

### Bisbenzimide assay

The bisbenzimide assay was performed by modifying the protocol by Roy Chowdhury *et al.* [93]. Briefly, overnight cultures of STM WT, STM Δ*pflB*, STM Δ*pflB*/pQE60: *pflB*, and STM Δ*fliC* were subcultured into fresh LB tubes(+/-formate). Logarithmic phase cultures of each condition were taken and the OD_600_ was adjusted to 0.1. To 180 µl of this bacterial suspension, 20 µl of bisbenzimide dye (10µg/ml) was added. It was incubated at 37˚C for 10 mins. The intracellular DNA-bound bisbenzimide was quantified in a TECAN 96 well microplate reader using a 346 nm excitation and 460 nm emission filter.

### Staining with DiBAC_4_

The DiBAC_4_ staining was performed by modifying the protocol by Roy Chowdhury *et al.* [97]. Membrane potential or membrane porosity was quantified by using a membrane potential sensitive fluorescent dye bis-(1,3-dibutyl barbituric acid)- trimethylene oxonol (Invitrogen). For this purpose, overnight culture of STM WT, STM Δ*pflB*, STM Δ*pflB*/pQE60: *pflB*, and STM Δ*fliC* were subcultured with or without the supplementation of 40 mM sodium formate. Around 4.5*10^7^ cells of the logarithmic phase culture were incubated with 1 µg/ml of DiBAC_4_ dye at 37˚C for 30 mins. The percentage of DiBAC_4_-positive cells and the Median Fluorescence Intensity (MFI) of DiBAC_4_ was determined by flow cytometry (BD FACSVerse by BD Biosciences-US).

### BCECF-AM staining

The BCECF-AM staining was performed by modifying the protocol by Roy Chowdhury *et al* [93]. The acidification of the cytosol with the change in pH of extracellular conditions was determined using a cell-permeable dual excitation ratiometric dye 2’,7’-Bis-(2-Carboxyethyl)-5- (and-6)-Carboxyfluorescein, Acetoxymethyl Ester (BCECF-AM). Briefly, the logarithmic phase cultures of STM WT, STM Δ*pflB*, STM Δ*pflB*/pQE60: *pflB*, (+/-formate) were resuspended in buffers of different pH (5,5.5,6,7). They were incubated for 2 hours at 37˚C before the addition of 1 mg/ml BCECF-AM to make a final concentration of 20 µM. The cells were incubated with the dye for 30 minutes. Post-incubation, the cells were analysed by flow cytometry (BD FACSVerse by BD Biosciences, US) with 488 nm and 405 nm excitation and 535 nm excitation channels. The MFI of the bacterial cells at 488 nm and 405 nm was obtained from BD FACSuite software and the ratio of 488/405 was utilized to determine the cytosolic acidification in response to changing pH conditions.

### Intracellular pH measurement by pHuji plasmid

STM WT and STM Δ*pflB* were transformed with the plasmid pBAD:pHuji (plasmid #61555) that encodes a pH-sensitive red fluorescent protein pHuji [98,99]. The logarithmic cultures of STM WT/pBAD:pHuji and STM Δ*pflB*/pBAD:pHuji (+/-formate) were pelleted and resuspended in phosphate buffers of pH 5, 6, 7, 8, 9 in the presence of 40 µM sodium benzoate to equilibrate the intracellular pH with the extracellular one. The fluorescence intensity of intracellular bacteria resuspended in buffers of varying pH was plotted against pH to generate a standard curve. Fluorescence readings from LB-grown cultures of STM WT/pBAD:pHuji and STM Δ*pflB*/pBAD:pHuji (with and without formate treatment) were then interpolated on this curve to determine the intracellular pH of the bacterial cells *in vitro*. Data has been presented both as bar graphs and heat map.

### Mice survival assay

6-8 weeks old male C57BL/6 mice were acquired from the specific-pathogen free conditions of Central Animal Facility, IISc. To draw a comparison between the mortality rate of WT and mutant strains, the mice (n = 10) were orally gavaged with 10^8^ cells from the stationary phase culture of each. After infection, mice were monitored every day for survival, and the data was represented as percent survival.

### Determination of bacterial burden in different organs

To understand the organ burden of the WT and mutant strain post-infection in mice, 10^7^ cells from the overnight culture of the bacterial strains were orally gavaged into 6–8 weeks old C57BL/6 mice. 5 days post-infection, the mice were ethically euthanized, and the liver and spleen were acquired. The organs were homogenized, and appropriate dilutions of the homogenate were plated on SS Agar. The data was represented was log_10_CFU/gm-wt.

### Invasion assay in mice

Invasion assay in mice was performed by modifying the protocol by Chandra *et al.* [100]. Briefly, 10^7^ cells from the overnight culture of the bacterial cells were orally gavaged into 6–8 weeks old male C57BL/6 mice. 6 hours post-infection, the mice were ethically euthanized, and the intestine was acquired. It was homogenized, and appropriate dilutions of the homogenate were plated on SS Agar. Corresponding fecal matter was also collected, and it was also plated post homogenization. The data was represented was log_10_CFU/gm-wt.

### Intraperitoneal infection of mice

Intraperitoneal infection of mice was performed with 10^3^ CFU from the overnight cultures of WT and mutant strains. 3 days post-infection, the mice were ethically euthanized, and the liver and the spleen were obtained. The organ burden of the bacterial strains was determined by the same protocol as above.

### Protein purification

The *rpoE* and *csrA* genes were synthesized by Twist Biosciences (California, USA) and cloned into the pET28a (+) expression vector between the NdeI and XhoI restriction sites, incorporating an N-terminal hexa-histidine tag for purification purposes.

For protein expression, *E. coli* BL21(DE3) cells were transformed with the respective recombinant plasmids. A single transformed colony was used to inoculate 10 ml of LB medium, which was incubated overnight at 37°C with shaking at 180 rpm as the primary culture. Subsequently, 1% of this overnight culture was used to inoculate 1 liter of LB medium, which was then grown at 37°C with shaking at 180 rpm until the OD₆₀₀ reached 0.6–0.8. Protein expression was induced using 0.3 mM IPTG for RpoE and 1 mM IPTG for CsrA, followed by incubation at 18°C for 20 hours with shaking at 180 rpm (for CsrA) or 100 rpm (for RpoE). The bacterial cells were harvested by centrifugation at 5000 rpm for 25 minutes at 4°C, and the resulting pellets were stored at −20°C for future use.

For lysis, the cell pellets were resuspended in 40 ml of sonication buffer (50 mM Tris, 300 mM NaCl, 10% glycerol, 10 mM imidazole, 2 mM β-mercaptoethanol, pH 7.5), with one Roche cOmplete™ Protease Inhibitor Cocktail tablet and 10 mM PMSF added to inhibit proteolytic degradation. Cells were lysed by sonication at 32% amplitude, with 3-second pulses followed by 6-second intervals. The lysate was then centrifuged at 13,000 rpm for 45 minutes at 4°C. The supernatant was filtered and applied to a His-Trap Ni^2+^ -NTA affinity column (Cytiva). After washing with buffer containing 20 mM imidazole, bound proteins were eluted using an elution buffer containing 300 mM imidazole.

Following affinity purification, proteins were concentrated to 4 ml using a Centricon device (Millipore) and subjected to size-exclusion chromatography (SEC) on an S75 Superdex column (Cytiva) equilibrated with SEC buffer (25 mM Tris, 150 mM NaCl, 10% glycerol, 1 mM DTT, pH 7.5). Fractions containing pure RpoE and CsrA proteins were collected, and sample purity was evaluated using glycine SDS-PAGE for RpoE and tricine SDS-PAGE for CsrA. Purified protein samples were stored at 4°C for later use.

### CD spectroscopy

CD spectroscopy was used to investigate the secondary structure and folding of RpoE and CsrA in the SEC buffer. CD spectra were recorded on a JASCO J-815 spectropolarimeter, using a 1 mm pathlength quartz cuvette (Hellma Analytics) at a constant temperature of 20°C. Spectral scans were conducted in the far-UV range (200–260 nm) with a scanning speed of 100 nm/min and a response time of 4 s. The sample concentration was set to 10 μM for all measurements. The data were averaged over three independent scans to improve accuracy, and baseline correction was applied to account for contributions from the buffer solution. The raw data (mDeg) was converted to molar ellipticity using the JASCO spectra analysis software.

### AlphaFold

FASTA sequences of RpoE and CsrA were provided as input sequences for Alphafold 3 (AF3) modelling of proteins. AF3 predictions were run using the available web server (https://alphafoldserver.com) [101]. The predicted models were visualized using PyMOL (DeLano Scientific).

### Isothermal titration calorimetry (ITC)

The ITC experiments were performed using the Malvern MIcroCal PEAQ-ITC machine at 25°C. Sodium formate (ligand) was solubilized in the SEC buffer of the protein samples (RpoE and CsrA). The titration was performed with 10 mM CsrA in the cell and 100 mM sodium formate in the syringe with 13 injections of 3 ml each and injection spacing of 150 s. The titration of RpoE was carried out at similar settings but with 20 mM protein in the cell and 200 mM sodium formate in the syringe. The integrated heat data was corrected for heat of dilution. Data was fitted to one site binding model, and the baseline was corrected and fit adjusted to minimize error using MicroCal PEAQ-ITC analysis software provided by the manufacturer.

### Kit based assay to determine bacterial intracellular formate

Caco-2 cells, seeded in 6-well plates, were infected with STM WT cultures in the logarithmic growth phase at a MOI of 50. The infection was allowed to proceed for 30 minutes, after which non-adherent and extracellular bacteria were removed by washing the cells twice with PBS. Eukaryotic cells were lysed using 0.1% Triton X-100, and the resulting lysate was centrifuged at 6,000 rpm and 4°C to selectively pellet the bacteria. The LB-grown cultures that had been used for infection were also centrifuged. The bacterial pellets were washed with 1X PBS to eliminate residual host cell debris or media debris, followed by sonication to lyse the bacterial cells. All procedures, except those involving cell culture, were conducted at 4°C. The resulting bacterial lysate was used to measure formate levels using a kit-based assay, following the manufacturer’s instructions (Abcam ab111748).

### *Salmonella* specific *pflB* primer designing

PRIMER-BLAST was used to design primers that specifically amplify the *pflB* gene from *Salmonella*. Additionally, using BLAST-n we assessed potential alignment matches for reverse complement sequence of the *pflB* RT reverse primer and observed up to 89% sequence similarity (S13 Data), primarily with plant pathogenic organisms. To rule out any chance of non-specific amplification within and below this similarity threshold, we analysed melt curves during qRT-PCR to confirm that the signal originated from a single, specific amplicon (S11B Fig).

### Statistical analysis

Each experiment has been repeated 1–6 times independently as mentioned in the figure legends. The statistical analyses were performed by unpaired Student’s t-test or two-way ANOVA multiple comparison test as denoted in the figure legends. The data obtained from the mice experiments were analyzed using the Mann-Whitney *U* test. The *p*-values below 0.05 were considered significant. The results are indicated as Mean ± SD or Mean ± SEM as mentioned in the figure legends. All the data were plotted and analysed using GraphPad Prism 8.4.2.

## Supporting information

S1 Fig STM Δ*pflB*, and STM Δ*focA* are not growth deficient in LB Broth, M9 Minimal Media. Deletion of pflB caused an enhanced expression of *focA.* A. Growth curves of STM WT, STM Δ*pflB* and STM Δ*focA* in LB broth. Data is represented as Mean + /-SEM of N = 2, n = 2. B. Growth curves of STM WT, STM Δ*pflB* and STM Δ*focA* in M9 Minimal Media. Data is represented as Mean + /-SEM of N = 2, n = 2. C. RT-qPCR mediated expression profile of *pflB* gene in the logarithmic cultures of STM Δ*focA*. Data is represented as Mean + /-SD of n = 3. D. RT-qPCR mediated expression profile of *focA* gene in the logarithmic cultures of STM Δ*pflB*. Data is represented as Mean + /-SD of n = 3. (Unpaired two-tailed Student’s t-test for column graphs, Two-way ANOVA for grouped data, Mann-Whitney U-test for animal experiment data (**** p < 0.0001, *** p < 0.001, ** p < 0.01, * p < 0.05)).
(TIF)

S2 Fig Intraperitoneal infection of STM WT and STM Δ*pflB* lead to no significant change in the bacterial burden in blood, liver, and spleen 3 dpi. A. Schematic showing the protocol followed for determining the organ burden of STM WT and STM Δ*pflB* 3 days post intraperitoneal infection B-D. Bacterial burden of STM WT and STM Δ*pflB* in blood (B), liver (C), and spleen (D). Data is represented as Mean + /-SEM of N = 2, n = 5. (Unpaired two-tailed Student’s t-test for column graphs, Two-way ANOVA for grouped data, Mann-Whitney U-test for animal experiment data (*** p < 0.0001, *** p < 0.001, ** p < 0.01, * p < 0.05)).
(TIF)

S3 Fig Intracellular and extracellular formate modulate the expression and secretion of SPI-1 arsenals in STM WT and STM Δ*pflB.* A. Expression of *hilA* in STM WT in formate supplemented at concentrations of 10, 20, 30, 40, and 50 mM. Data is representative of N = 2, n = 3 and expressed as Mean + /- SD. B. Representative histograms of MFI of APC (reflecting SipC expression) in FITC positive (bacteria-infected) Caco-2 cells upon infection with STM WT, STM Δ*pflB*, STM Δ*pflB/*pQE60:*pflB* (+/-F), STM Δ*rpoE*, STM Δ*pflB*Δ*rpoE,* STM Δ*sirA*, STM Δ*pflB*Δ*sirA* (+/-F). Data is representative of N = 3, n ≥ 4. (Unpaired two-tailed Student’s t-test for column graphs, Two-way ANOVA for grouped data, Mann-Whitney U-test for animal experiment data (**** p < 0.0001, *** p < 0.001, ** p < 0.01, * p < 0.05)).
(TIF)

S4 Fig Confocal microscopy assisted visualization of the bacterial adhesion and RT-qPCR mediated assessment of the concentration-dependent effects of formate treatment on the expression of *fliC* and *hilA* in WT and *pflB* knocked out *Salmonella.* A. Adhesion assay performed on Caco-2 cells and visualized by confocal microscopy. Data is representative of N = 2, n ≥ 10 B. Expression of *fliC* in STM WT and STM Δ*pflB* in formate supplemented at concentrations of 0.067, 0.125, 0.5, 1, 10 mM. Data is represented as Mean + /-SEM of N = 4, n = 3. C. Expression of *hilA* in STM WT and STM Δ*pflB* in formate supplemented at concentrations of 0.067, 0.125, 0.5, 1, 10 mM. Data is represented as Mean + /-SEM of N = 4, n = 3. (Unpaired two-tailed Student’s t-test for column graphs, Two-way ANOVA for grouped data, Mann-Whitney U-test for animal experiment data (**** p < 0.0001, *** p < 0.001, ** p < 0.01, * p < 0.05)).
(TIF)

S5 Fig Confocal microscopy assisted visualization of intracellular bacteria upon infection of STM WT, STM Δ*pflB* (+/-F), STM Δ*rpoE*, and STM Δ*pflB*Δ*rpoE.* Figure showing the invaded bacteria in Caco-2 upon infection with STM WT, STM Δ*pflB* (+/-F), STM Δ*rpoE*, and STM Δ*pflB*Δ*rpoE*. Some bacteria might appear pixelated due to their localization in different focal planes. Data is representative of N = 2, n ≥ 50.
(TIF)

S6 Fig STM Δ*focA*Δ*pflB* had an attenuated adhesion, with partial recovery upon supplementation of extragenous F. A. Growth curves of STM WT, STM Δ*focA*Δ*pflB* in LB broth and M9 Minimal Media. Data is represented as Mean + /-SEM of N = 2, n = 2. B. Growth curves of STM WT, STM Δ*pflB* and STM Δ*focA* in M9 Minimal Media. Data is represented as Mean + /-SEM of N = 2, n = 2. C. Percent adhesion of STM WT and STM Δ*focA*Δ*pflB* (+/-F) in Caco-2 cell line. Data is represented as Mean + /-SEM of N = 5, n = 3. D. Organ burden of STM WT, STM Δ*focA* and STM Δ*focA*Δ*pflB* in liver of C57BL/6 mice 5 days post oral gavaging. Data is represented as Mean + /-SEM of N = 2, n = 5. E. Organ burden of STM WT, STM Δ*focA* and STM Δ*focA*Δ*pflB* in spleen of C57BL/6 mice 5 days post oral gavaging. Data is represented as Mean + /-SEM of N = 2, n = 5. (Unpaired two-tailed Student’s t-test for column graphs, Two-way ANOVA for grouped data, Mann-Whitney U-test for animal experiment data (**** p < 0.0001, *** p < 0.001, ** p < 0.01, * p < 0.05)).
(TIF)

S7 Fig Increased membrane depolarization in STM Δ*pflB* occurred independently of its flagellar deficiency. A. Representative FACS profiles showing the percentage of DiBAC_4_-positive cells in logarithmic-phase cultures of STM WT, STM Δ*pflB*, and STM Δ*fliC* (+/- F) grown in LB and F-media. Data is representative of N = 4, n = 3. B. Flow cytometry-based quantification of DiBAC_4_-positive cells (%) in logarithmic-phase cultures of STM WT, STM Δ*pflB*, and STM Δ*fliC* (+/-F) incubated in LB (pH 7.2) and F-media (pH 5.5). Data are shown as mean + /- SEM from N = 4, n = 3. C. Bisbenzimide assay-based quantification of membrane damage in logarithmic-phase cultures of STM WT, STM Δ*pflB*, and STM Δ*fliC* (+/- F). Data is represented as mean ± SEM from N = 6, n = 5. (Unpaired two-tailed Student’s t-test for column graphs, Two-way ANOVA for grouped data, Mann-Whitney U-test for animal experiment data (**** p < 0.0001, *** p < 0.001, ** p < 0.01, * p < 0.05)).
(TIF)

S8 Fig Decreased expression of *fliC* and *csrA* in STM *ΔpflB* can be altered upon incubating the bacterial cultures in acidic media (pH of 6). A. RT-qPCR mediated expression profile of *fliC* in the logarithmic cultures of STM WT, STM Δ*pflB* incubated in acidic LB media (pH 6). Data is represented as Mean + /-SEM of N = 3, n = 3. B. RT-qPCR mediated expression profile of *csrA* in the logarithmic cultures of STM WT, STM Δ*pflB* incubated in acidic LB media (pH 6). Data is represented as Mean + /-SEM of N = 3, n = 3. (Unpaired two-tailed Student’s t-test for column graphs, Two-way ANOVA for grouped data, Mann-Whitney U-test for animal experiment data (**** p < 0.0001, *** p < 0.001, ** p < 0.01, * p < 0.05)).
(TIF)

S9 Fig Purification and structural characterization of CsrA and RpoE. A. Figure panel showing SEC elution profile of CsrA. Protein eluted as an approximate dimer. B. Panel showing tricine SDS-PAGE analysis of the purified CsrA after SEC purification. C. CD spectra of CsrA D. AlphaFold 3 model of CsrA E. SEC elution profile of RpoE. Protein eluted as an approximate monomer. F. A glycine SDS-PAGE analysis of the purified RpoE after SEC purification G. CD spectra of RpoE H. AlphaFold 3 model of RpoE.
(TIF)

S10 Fig Compensation of intracellular formate pool in STM Δ*pflB* makes it mimic the behaviour of STM WT. TEM assisted visualization of the flagellar structures in STM WT and STM *ΔpflB* supplemented with 40, 90, 110 mM of sodium formate. Data is representative of N = 2, n = 10.
(TIF)

S11 Fig Three-dimensional visualization of immunohistochemistry images illustrating spatial localization of SipC. A. Melt curve analysis of amplicons generated using *Salmonella*-specific *pflB* primers. B. Three-dimensional representation of immunohistochemistry images from duodenal and ileal sections infected with STM WT and STM Δ*pflB*. Images are representative of n = 7.
(TIF)

S12 Fig Graphical abstract: A schematic illustrating the regulation of flagellar and SPI-1 genes by coordination of intracellular and extracellular formate. (TIF)

S13 Data BLAST-n analysis of *pflB* RT primers. (CSV)
