# Supplementary figures and images for: Intracellular formate modulates a motility-invasion switch in Salmonella Typhimurium
Source: PLoS Pathog. 2025 Sep 4;21(9):e1013453. doi: 10.1371/journal.ppat.1013453 (PMC12410710; doi:10.1371/journal.ppat.1013453)

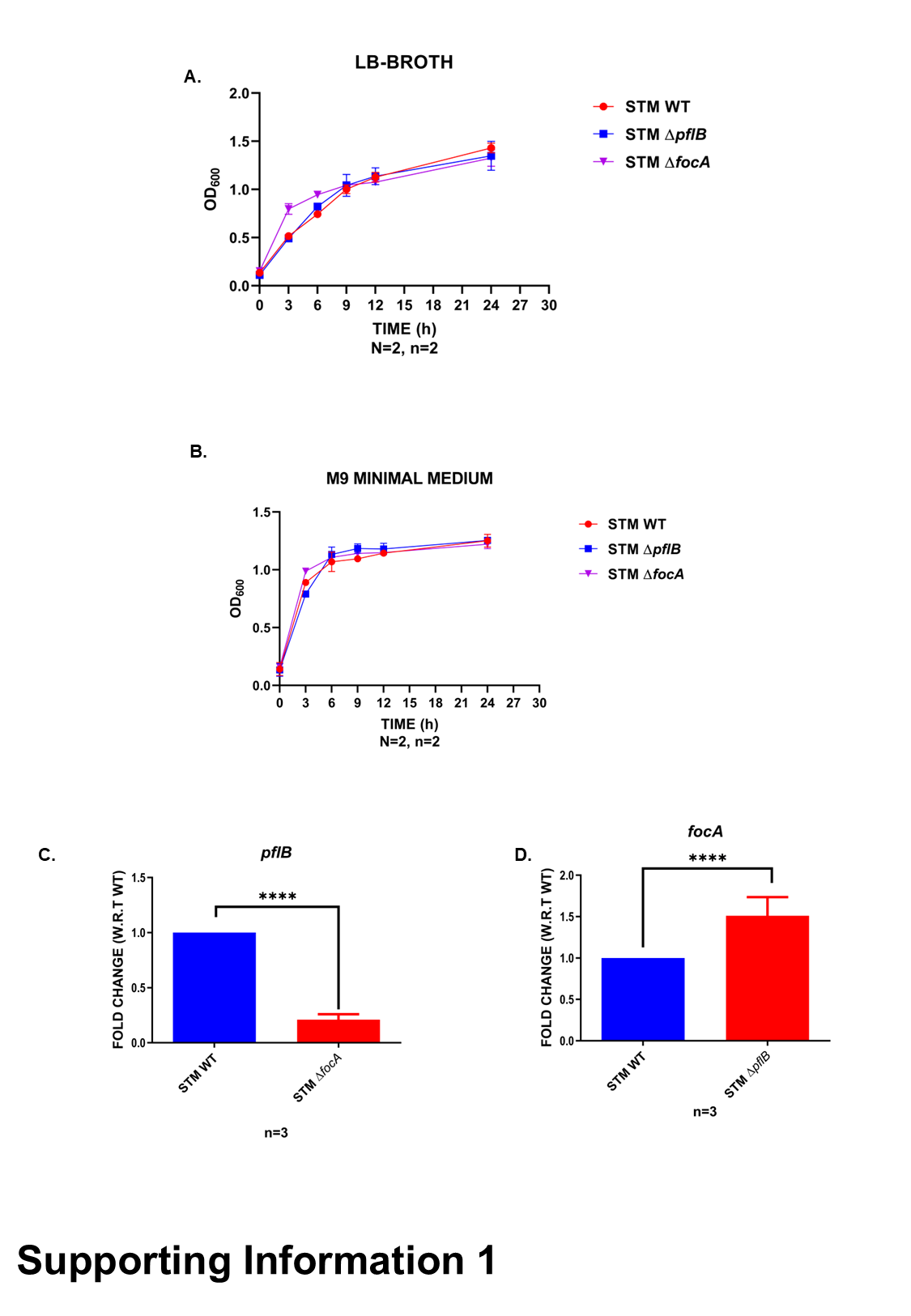

Supplement: S1 Fig — Deletion of pflB caused an enhanced expression of focA. A. Growth curves of STM WT, STM ΔpflB and STM ΔfocA in LB broth. Data is represented as Mean + /-SEM of N = 2, n = 2. B. Growth curves of STM WT, STM ΔpflB and STM ΔfocA in M9 Minimal Media. Data is represented as Mean + /-SEM of N = 2, n = 2. C. RT-qPCR mediated expression profile of pflB gene in the logarithmic cultures of STM ΔfocA. Data is represented as Mean + /-SD of n = 3. D. RT-qPCR mediated expression profile of focA gene in the logarithmic cultures of STM ΔpflB. Data is represented as Mean + /-SD of n = 3. (Unpaired two-tailed Student’s t-test for column graphs, Two-way ANOVA for grouped data, Mann-Whitney U-test for animal experiment data (**** p < 0.0001, *** p < 0.001, ** p < 0.01, * p < 0.05)). (TIF) [file ppat.1013453.s001.TIF]

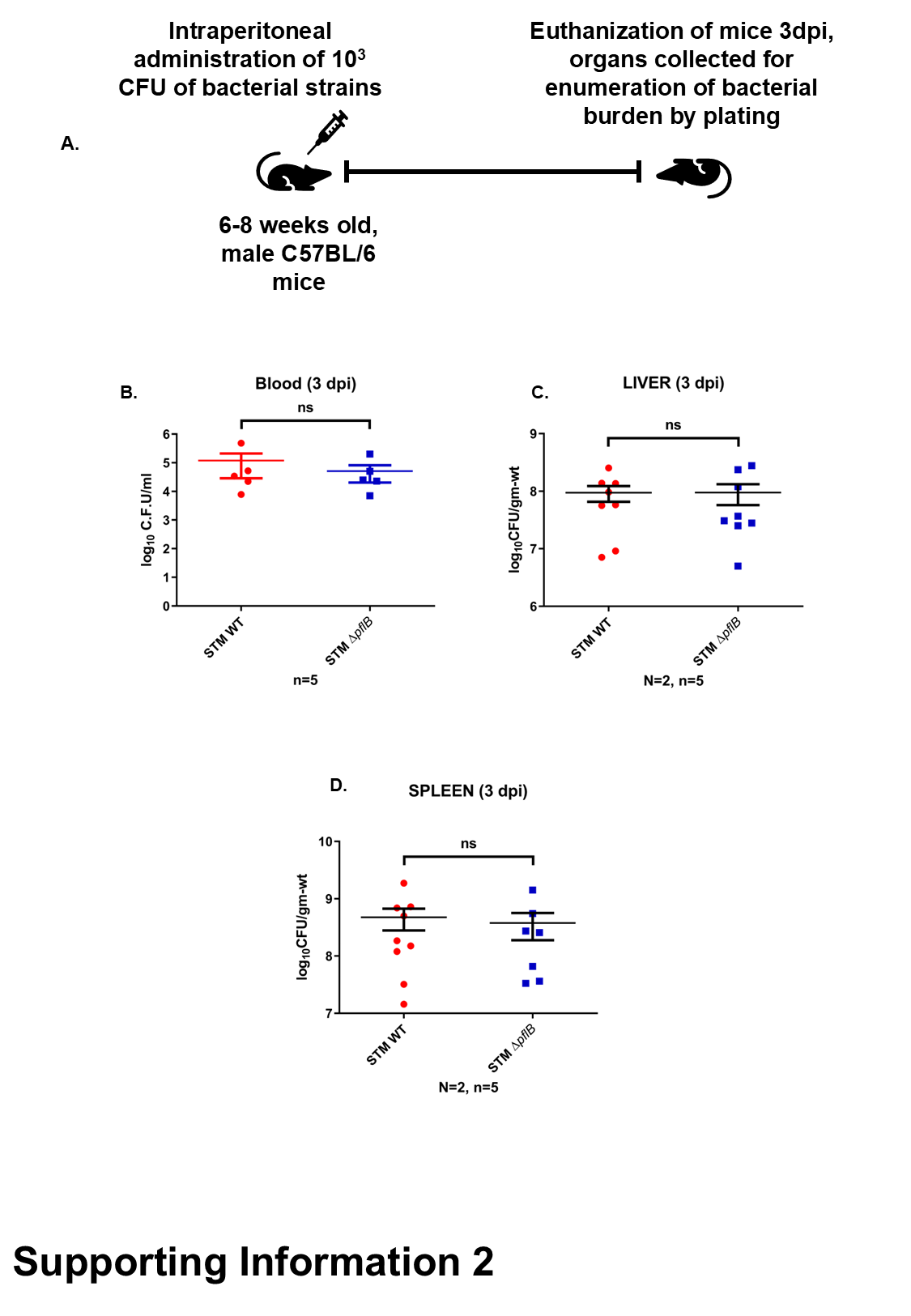

Supplement: S2 Fig — A. Schematic showing the protocol followed for determining the organ burden of STM WT and STM ΔpflB 3 days post intraperitoneal infection B-D. Bacterial burden of STM WT and STM ΔpflB in blood (B), liver (C), and spleen (D). Data is represented as Mean + /-SEM of N = 2, n = 5. (Unpaired two-tailed Student’s t-test for column graphs, Two-way ANOVA for grouped data, Mann-Whitney U-test for animal experiment data (*** p < 0.0001, *** p < 0.001, ** p < 0.01, * p < 0.05)). (TIF) [file ppat.1013453.s002.TIF]

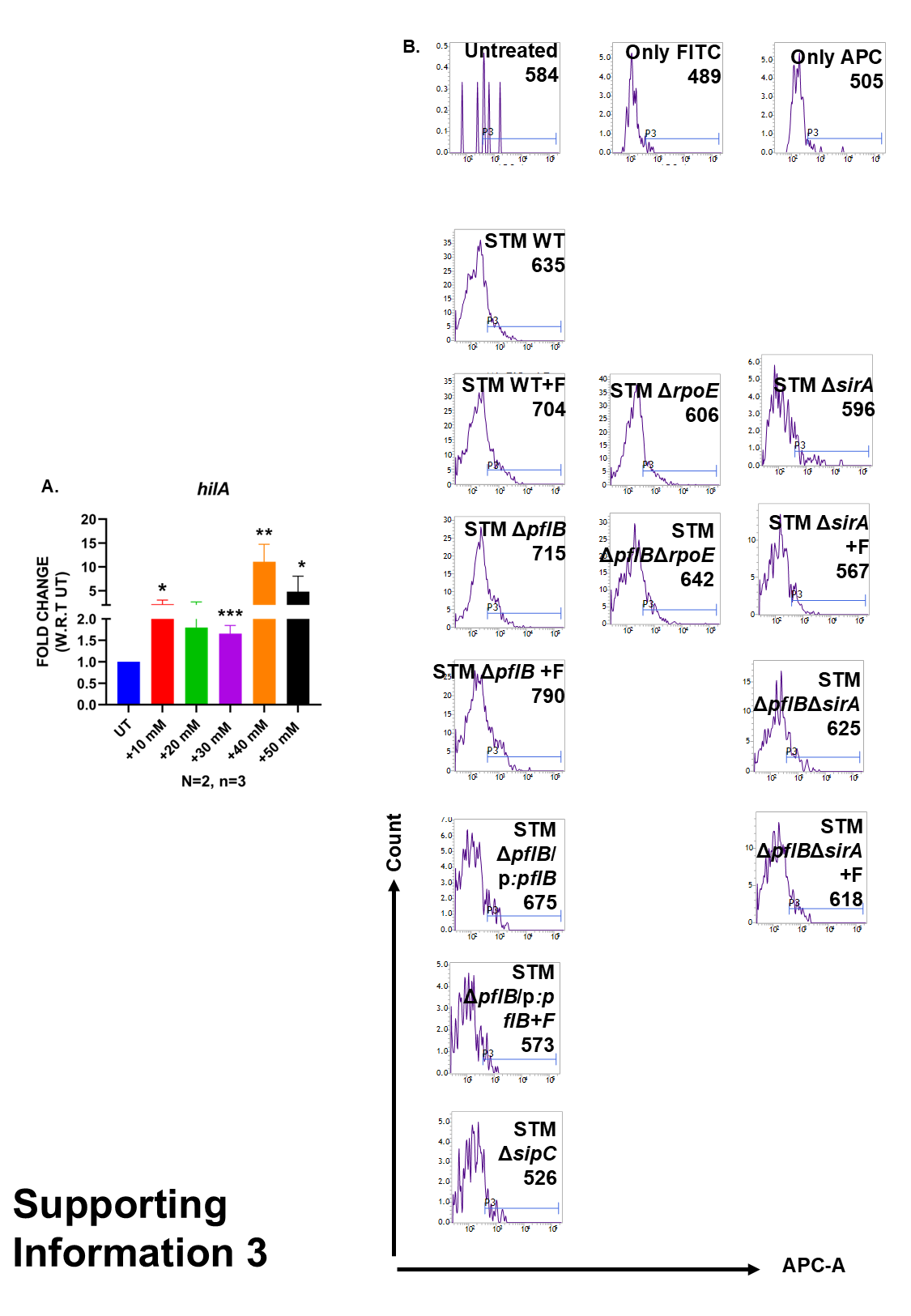

Supplement: S3 Fig — A. Expression of hilA in STM WT in formate supplemented at concentrations of 10, 20, 30, 40, and 50 mM. Data is representative of N = 2, n = 3 and expressed as Mean + /- SD. B. Representative histograms of MFI of APC (reflecting SipC expression) in FITC positive (bacteria-infected) Caco-2 cells upon infection with STM WT, STM ΔpflB, STM ΔpflB/pQE60:pflB (+/-F), STM ΔrpoE, STM ΔpflBΔrpoE, STM ΔsirA, STM ΔpflBΔsirA (+/-F). Data is representative of N = 3, n ≥ 4. (Unpaired two-tailed Student’s t-test for column graphs, Two-way ANOVA for grouped data, Mann-Whitney U-test for animal experiment data (**** p < 0.0001, *** p < 0.001, ** p < 0.01, * p < 0.05)). (TIF) [file ppat.1013453.s003.TIF]

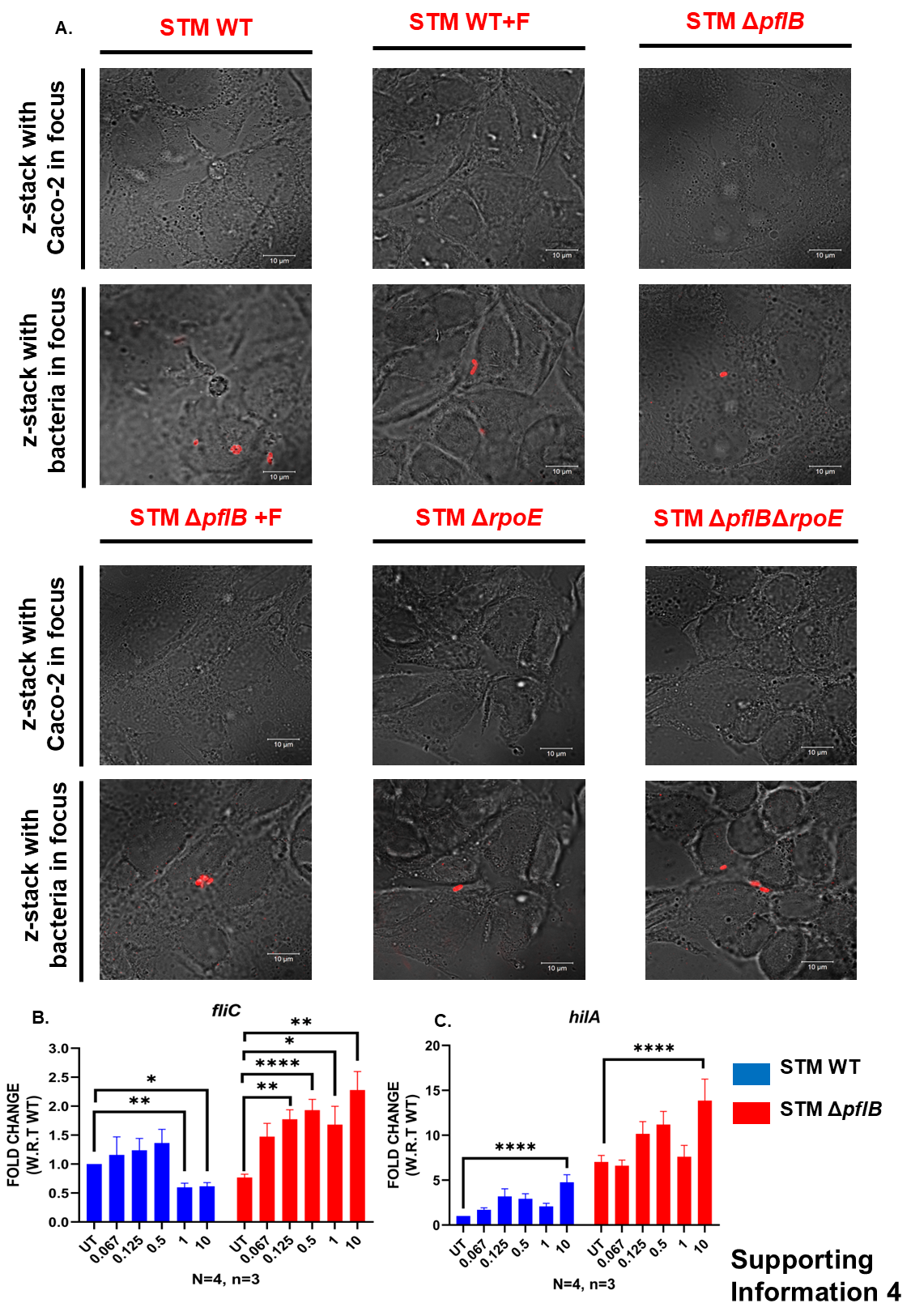

Supplement: S4 Fig — A. Adhesion assay performed on Caco-2 cells and visualized by confocal microscopy. Data is representative of N = 2, n ≥ 10 B. Expression of fliC in STM WT and STM ΔpflB in formate supplemented at concentrations of 0.067, 0.125, 0.5, 1, 10 mM. Data is represented as Mean + /-SEM of N = 4, n = 3. C. Expression of hilA in STM WT and STM ΔpflB in formate supplemented at concentrations of 0.067, 0.125, 0.5, 1, 10 mM. Data is represented as Mean + /-SEM of N = 4, n = 3. (Unpaired two-tailed Student’s t-test for column graphs, Two-way ANOVA for grouped data, Mann-Whitney U-test for animal experiment data (**** p < 0.0001, *** p < 0.001, ** p < 0.01, * p < 0.05)). (TIF) [file ppat.1013453.s004.tif]

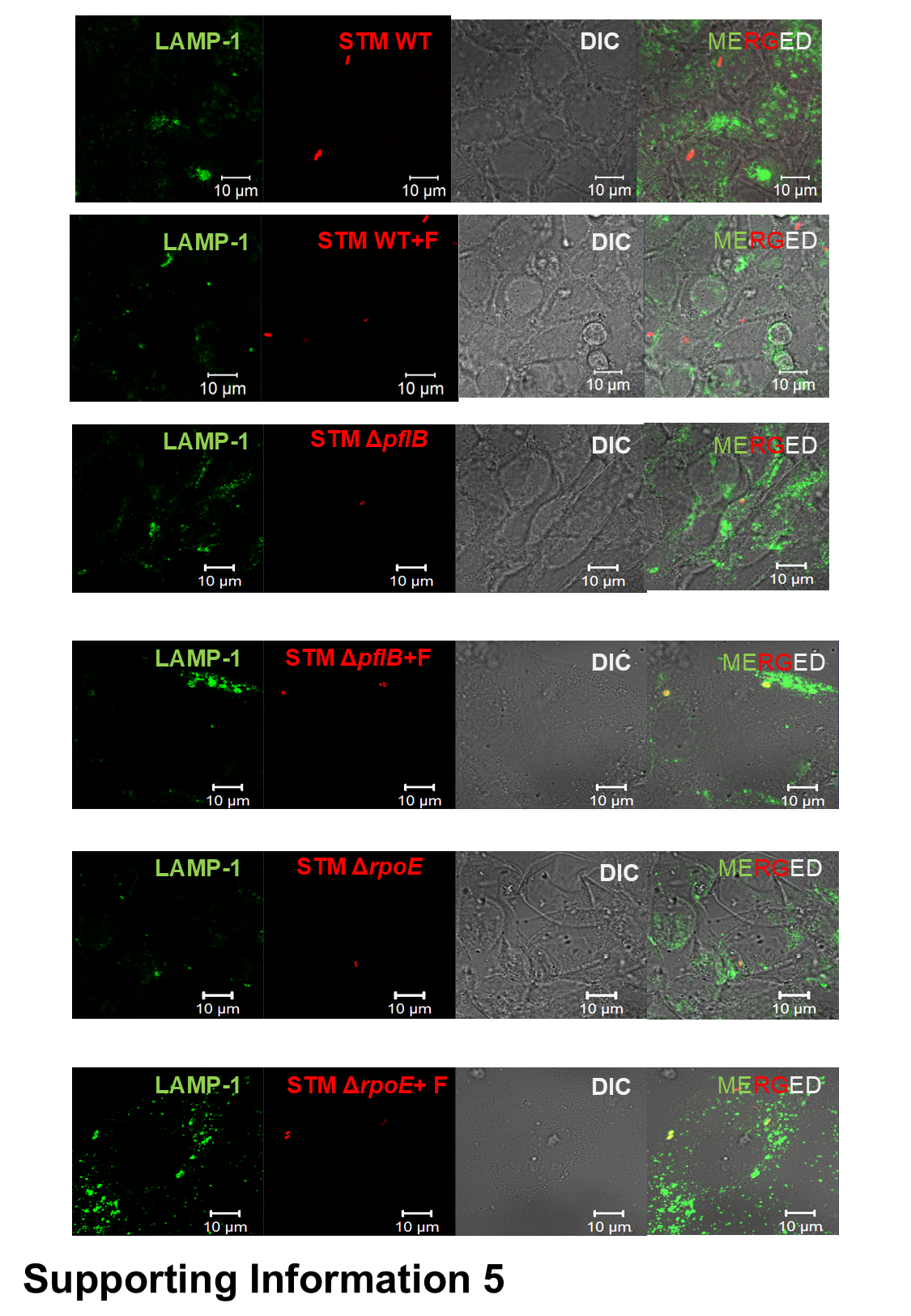

Supplement: S5 Fig — Figure showing the invaded bacteria in Caco-2 upon infection with STM WT, STM ΔpflB (+/-F), STM ΔrpoE, and STM ΔpflBΔrpoE. Some bacteria might appear pixelated due to their localization in different focal planes. Data is representative of N = 2, n ≥ 50. (TIF) [file ppat.1013453.s005.TIF]

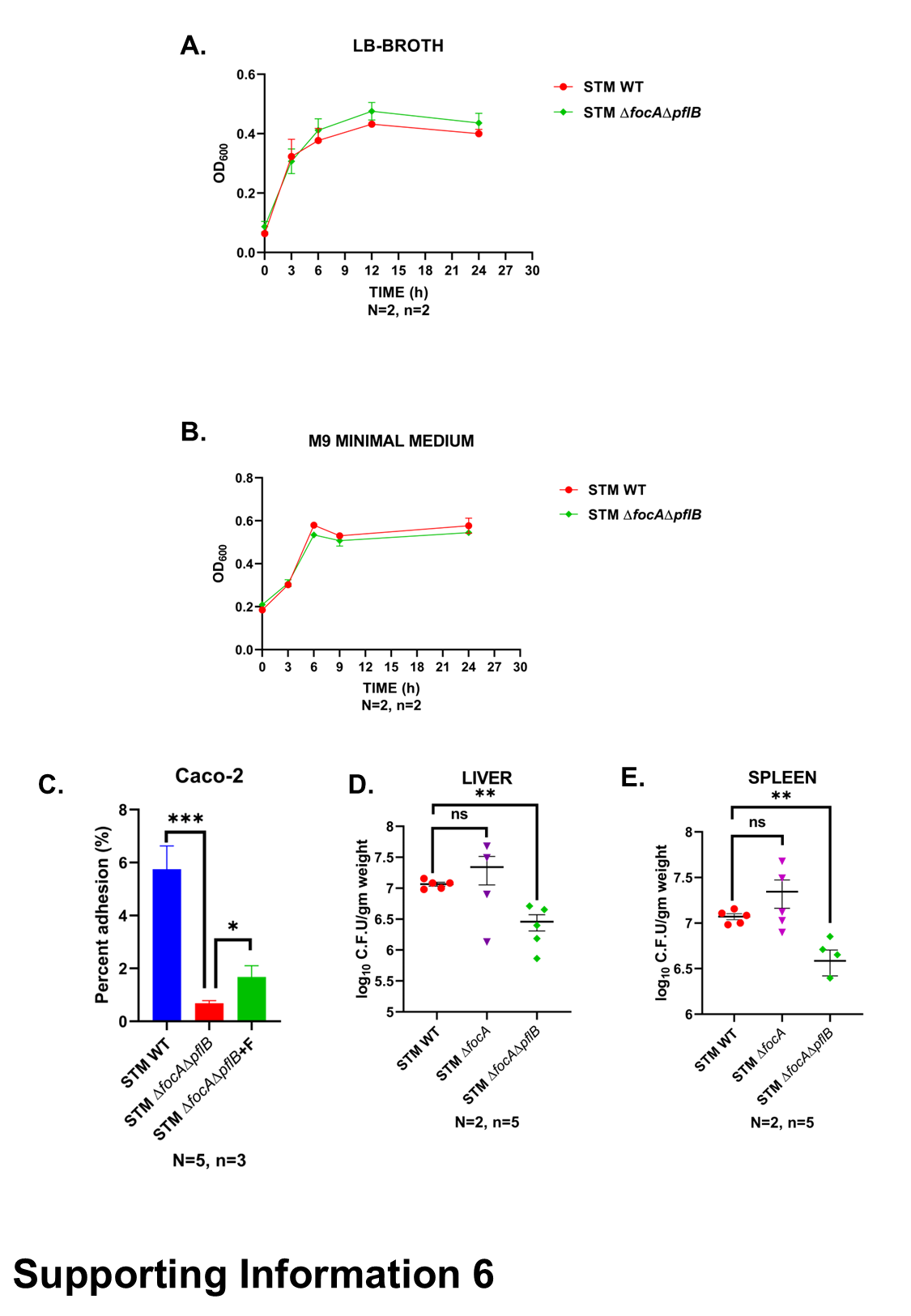

Supplement: S6 Fig — A. Growth curves of STM WT, STM ΔfocAΔpflB in LB broth and M9 Minimal Media. Data is represented as Mean + /-SEM of N = 2, n = 2. B. Growth curves of STM WT, STM ΔpflB and STM ΔfocA in M9 Minimal Media. Data is represented as Mean + /-SEM of N = 2, n = 2. C. Percent adhesion of STM WT and STM ΔfocAΔpflB (+/-F) in Caco-2 cell line. Data is represented as Mean + /-SEM of N = 5, n = 3. D. Organ burden of STM WT, STM ΔfocA and STM ΔfocAΔpflB in liver of C57BL/6 mice 5 days post oral gavaging. Data is represented as Mean + /-SEM of N = 2, n = 5. E. Organ burden of STM WT, STM ΔfocA and STM ΔfocAΔpflB in spleen of C57BL/6 mice 5 days post oral gavaging. Data is represented as Mean + /-SEM of N = 2, n = 5. (Unpaired two-tailed Student’s t-test for column graphs, Two-way ANOVA for grouped data, Mann-Whitney U-test for animal experiment data (**** p < 0.0001, *** p < 0.001, ** p < 0.01, * p < 0.05)). (TIF) [file ppat.1013453.s006.TIF]

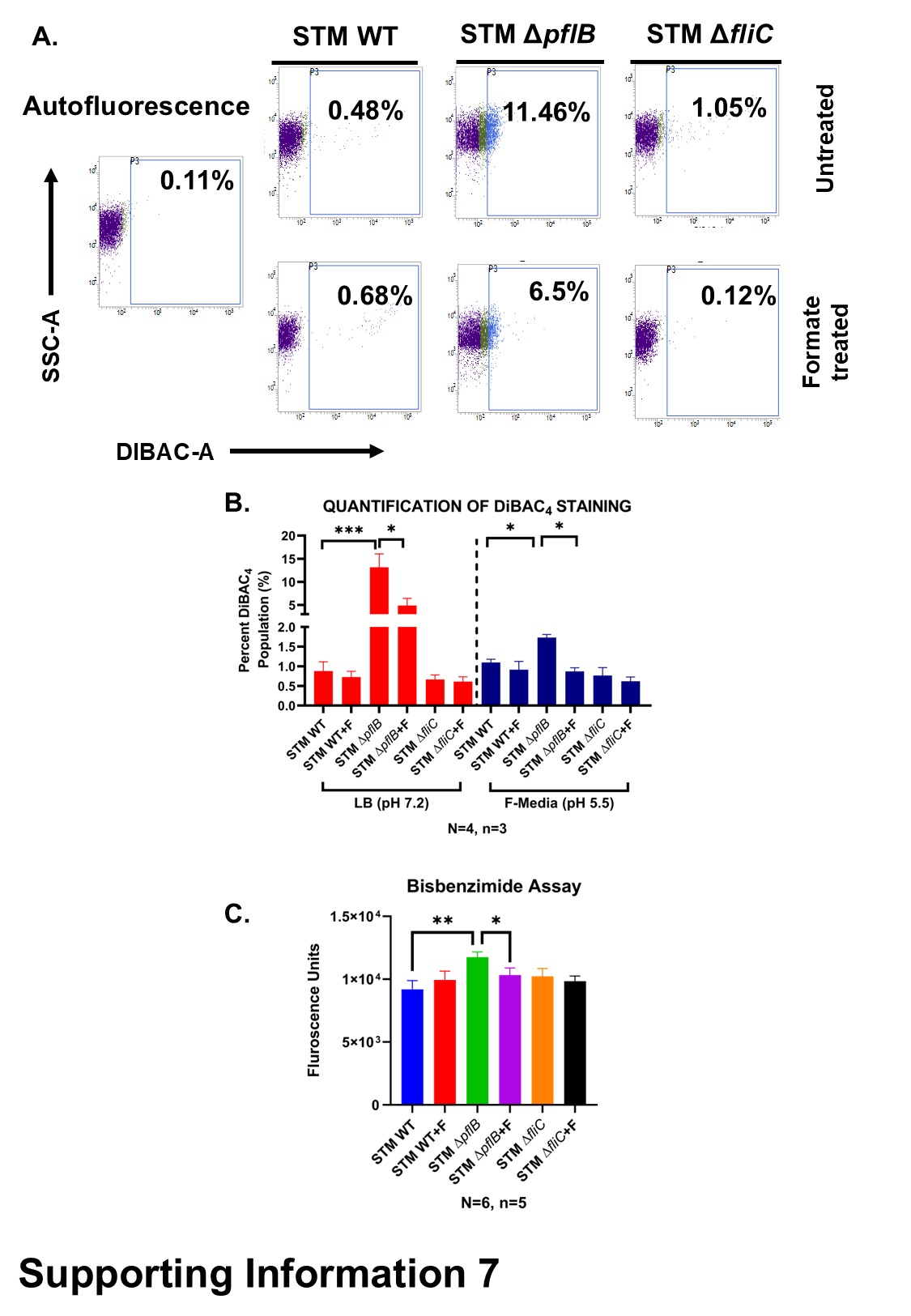

Supplement: S7 Fig — A. Representative FACS profiles showing the percentage of DiBAC4-positive cells in logarithmic-phase cultures of STM WT, STM ΔpflB, and STM ΔfliC (+/- F) grown in LB and F-media. Data is representative of N = 4, n = 3. B. Flow cytometry-based quantification of DiBAC4-positive cells (%) in logarithmic-phase cultures of STM WT, STM ΔpflB, and STM ΔfliC (+/-F) incubated in LB (pH 7.2) and F-media (pH 5.5). Data are shown as mean + /- SEM from N = 4, n = 3. C. Bisbenzimide assay-based quantification of membrane damage in logarithmic-phase cultures of STM WT, STM ΔpflB, and STM ΔfliC (+/- F). Data is represented as mean ± SEM from N = 6, n = 5. (Unpaired two-tailed Student’s t-test for column graphs, Two-way ANOVA for grouped data, Mann-Whitney U-test for animal experiment data (**** p < 0.0001, *** p < 0.001, ** p < 0.01, * p < 0.05)). (TIF) [file ppat.1013453.s007.TIF]

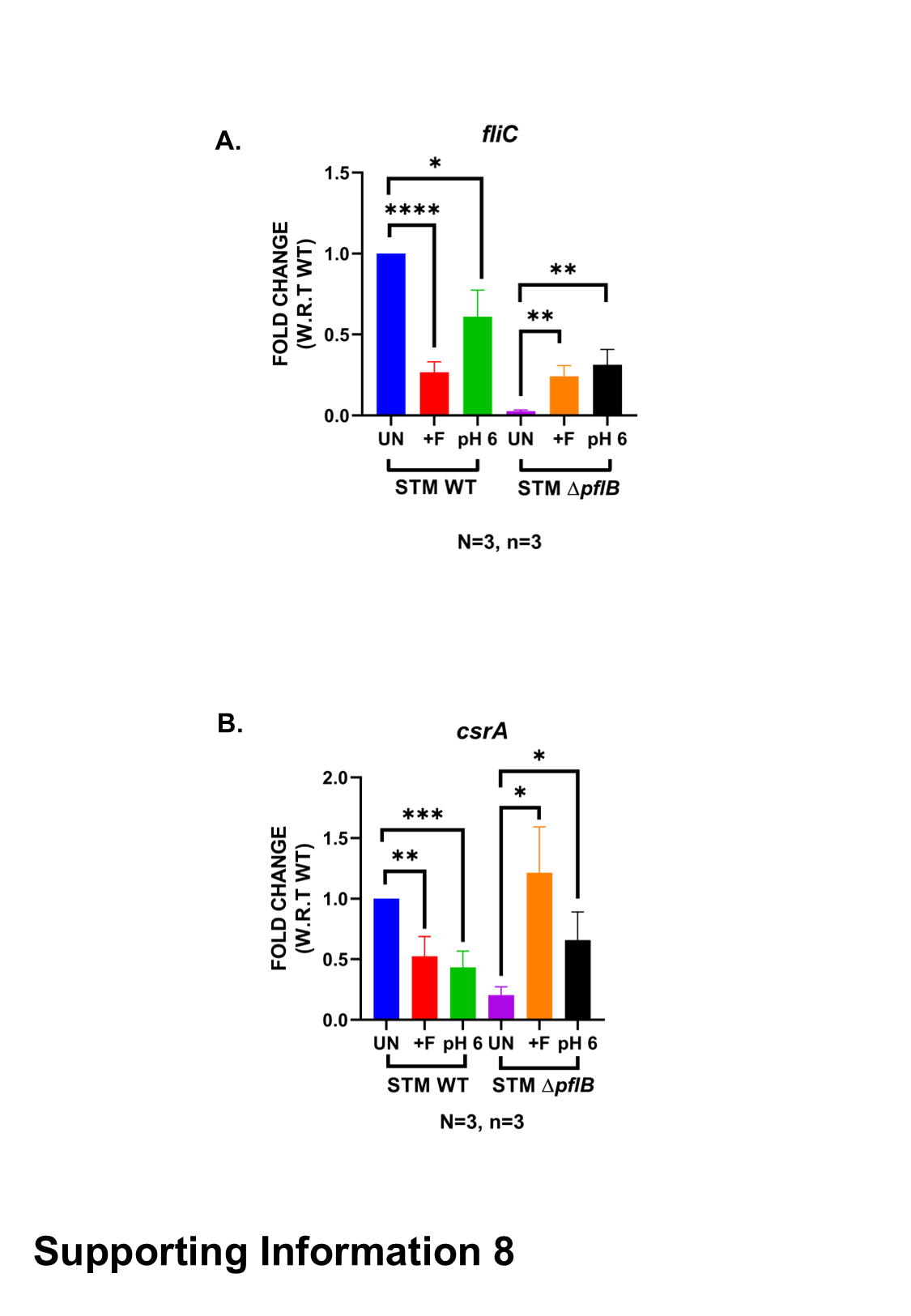

Supplement: S8 Fig — A. RT-qPCR mediated expression profile of fliC in the logarithmic cultures of STM WT, STM ΔpflB incubated in acidic LB media (pH 6). Data is represented as Mean + /-SEM of N = 3, n = 3. B. RT-qPCR mediated expression profile of csrA in the logarithmic cultures of STM WT, STM ΔpflB incubated in acidic LB media (pH 6). Data is represented as Mean + /-SEM of N = 3, n = 3. (Unpaired two-tailed Student’s t-test for column graphs, Two-way ANOVA for grouped data, Mann-Whitney U-test for animal experiment data (**** p < 0.0001, *** p < 0.001, ** p < 0.01, * p < 0.05)). (TIF) [file ppat.1013453.s008.TIF]

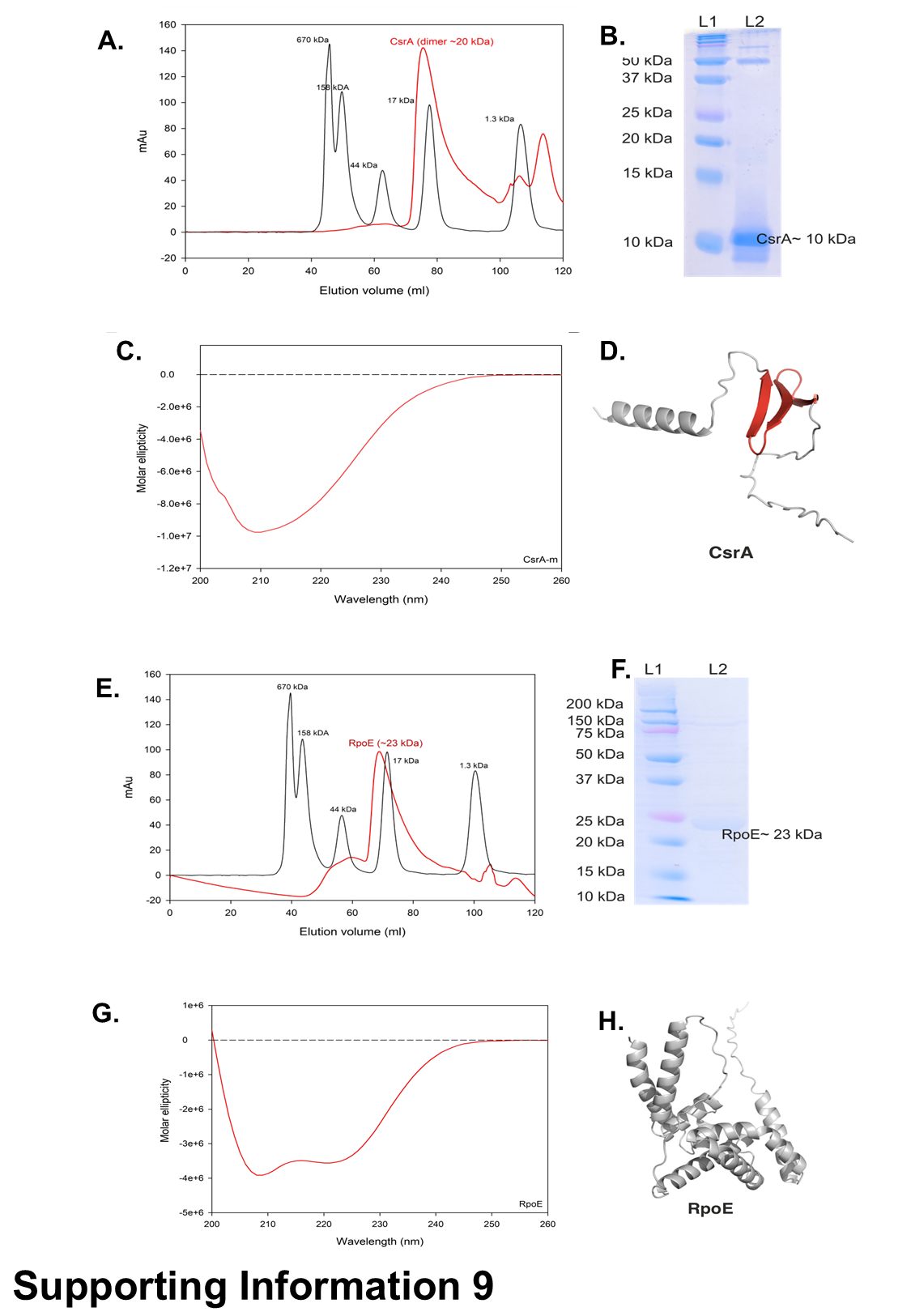

Supplement: S9 Fig — A. Figure panel showing SEC elution profile of CsrA. Protein eluted as an approximate dimer. B. Panel showing tricine SDS-PAGE analysis of the purified CsrA after SEC purification. C. CD spectra of CsrA D. AlphaFold 3 model of CsrA E. SEC elution profile of RpoE. Protein eluted as an approximate monomer. F. A glycine SDS-PAGE analysis of the purified RpoE after SEC purification G. CD spectra of RpoE H. AlphaFold 3 model of RpoE. (TIF) [file ppat.1013453.s009.TIF]

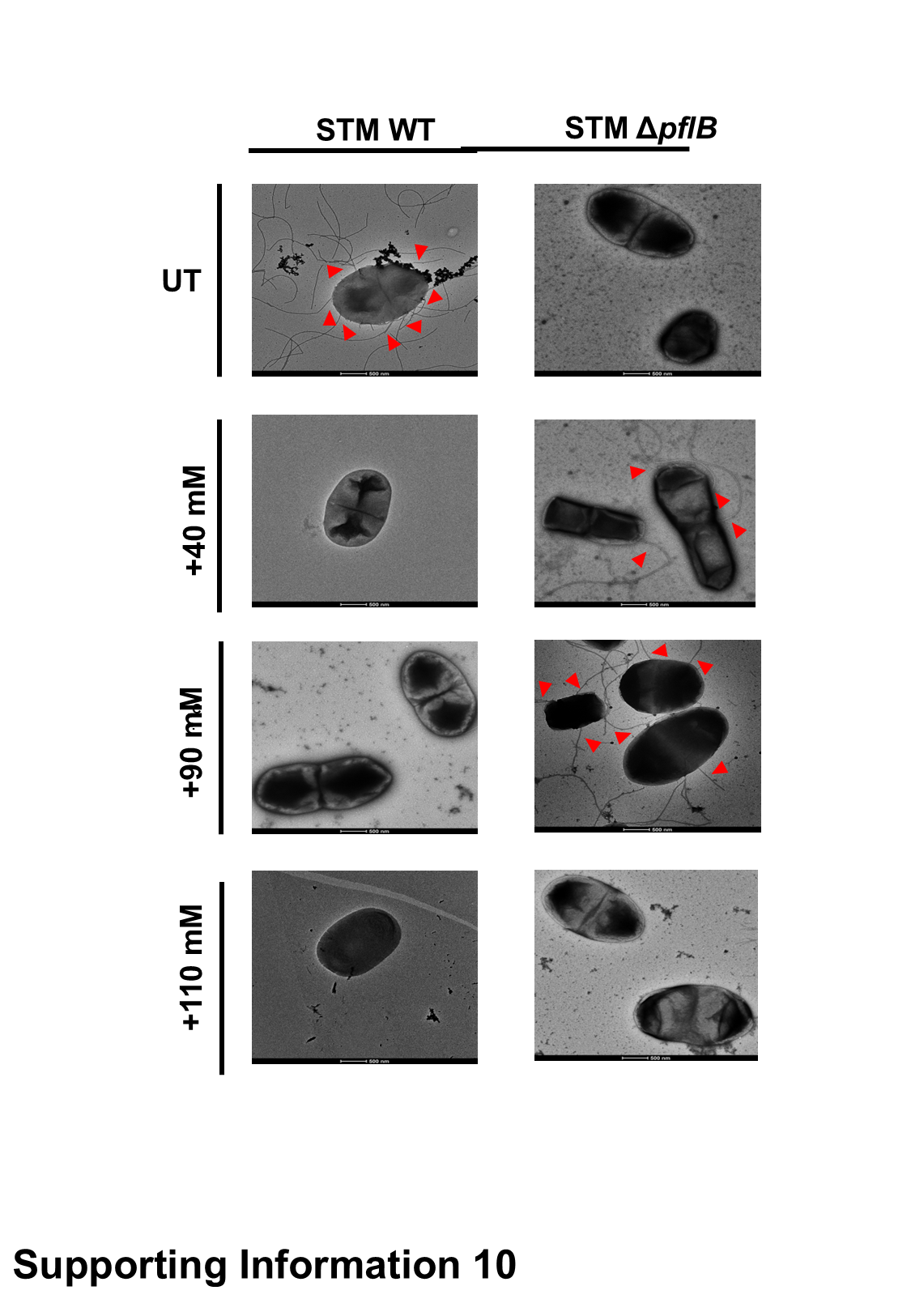

Supplement: S10 Fig — TEM assisted visualization of the flagellar structures in STM WT and STM ΔpflB supplemented with 40, 90, 110 mM of sodium formate. Data is representative of N = 2, n = 10. (TIF) [file ppat.1013453.s010.TIF]

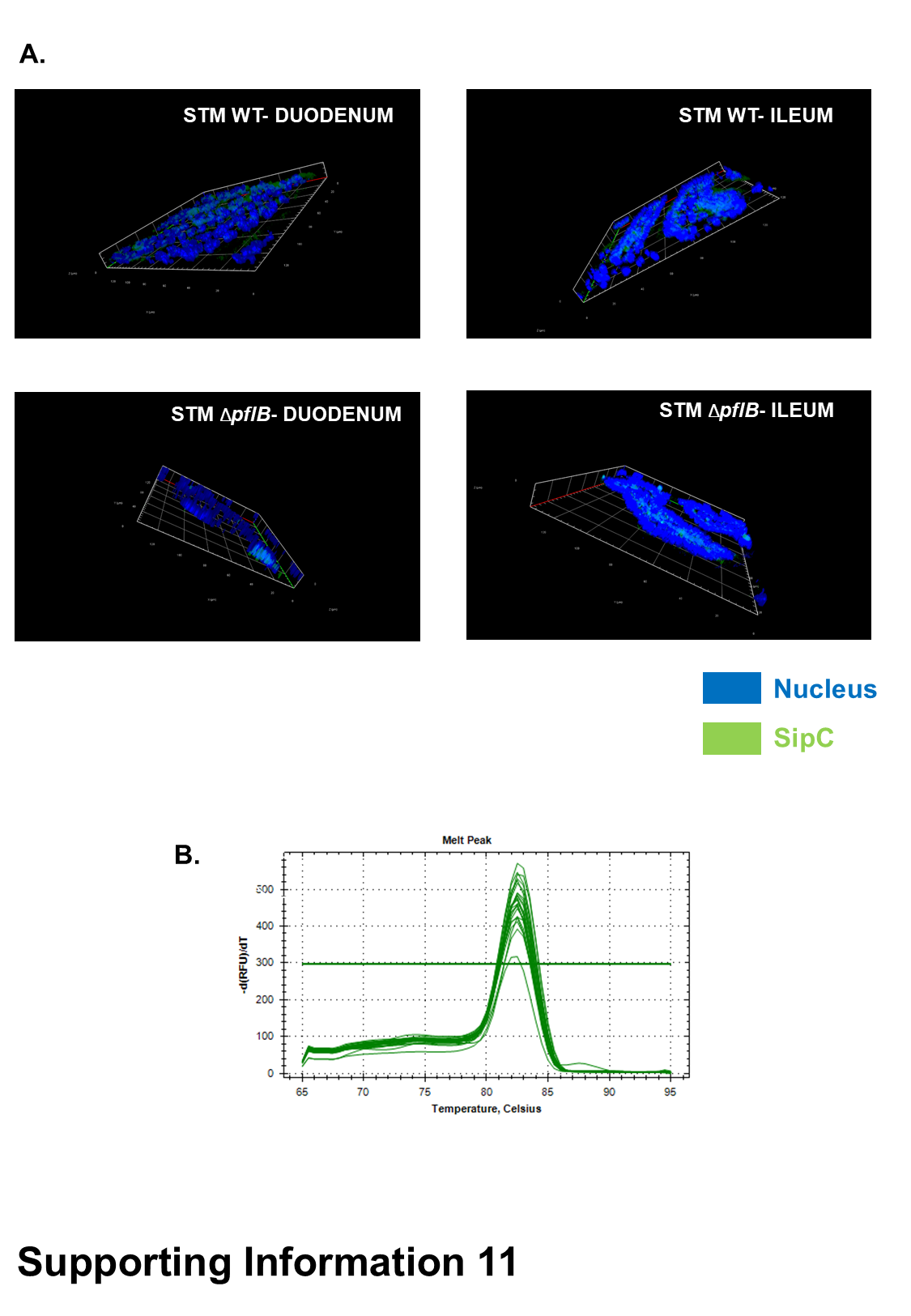

Supplement: S11 Fig — A. Melt curve analysis of amplicons generated using Salmonella-specific pflB primers. B. Three-dimensional representation of immunohistochemistry images from duodenal and ileal sections infected with STM WT and STM ΔpflB. Images are representative of n = 7. (TIF) [file ppat.1013453.s011.TIF]

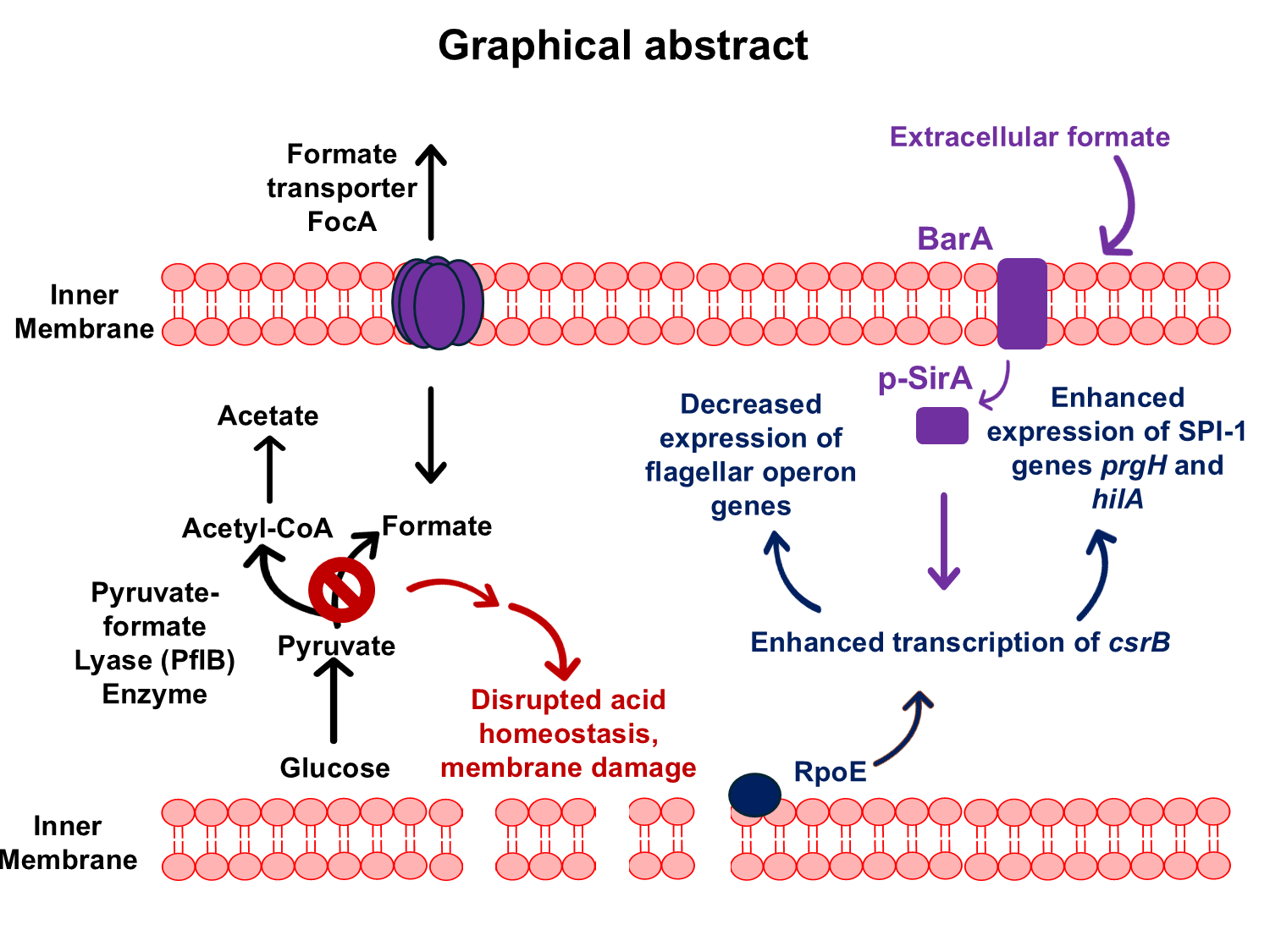

Supplement: S12 Fig — (TIF) [file ppat.1013453.s012.tif]
